# Scaling up of a Self‐Confined Catalytic Hybridization Circuit for Robust microRNA Imaging

**DOI:** 10.1002/advs.202400517

**Published:** 2024-04-13

**Authors:** Xue Gong, Ruomeng Li, Jiajia Zhang, Pu Zhang, Zhongwei Jiang, Lianzhe Hu, Xiaoqing Liu, Yi Wang, Fuan Wang

**Affiliations:** ^1^ Department of Gastroenterology Zhongnan Hospital of Wuhan University College of Chemistry and Molecular Sciences Wuhan University Wuhan 430072 P. R. China; ^2^ Engineering Research Center for Biotechnology of Active Substances (Ministry of Education) Chongqing Key Laboratory of Green Catalysis Materials and Technology College of Chemistry Chongqing Normal University Chongqing 401331 P. R. China; ^3^ College of Pharmacy Chongqing Medical University Chongqing 400016 P. R. China

**Keywords:** catalytic DNA circuit, diagnosis, DNA nanotechnology, imaging, self‐assembly

## Abstract

The precise regulation of cellular behaviors within a confined, crowded intracellular environment is highly amenable in diagnostics and therapeutics. While synthetic circuitry system through a concatenated chemical reaction network has rarely been reported to mimic dynamic self‐assembly system. Herein, a catalytic self‐defined circuit (CSC) for the hierarchically concatenated assembly of DNA domino nanostructures is engineered. By incorporating pre‐sealed symmetrical fragments into the preying hairpin reactants, the CSC system allows the hierarchical DNA self‐assembly via a microRNA (miRNA)‐powered self‐sorting catalytic hybridization reaction. With minimal strand complexity, this self‐sustainable CSC system streamlined the circuit component and achieved localization‐intensified cascaded signal amplification. Profiting from the self‐adaptively concatenated hybridization reaction, a reliable and robust method has been achieved for discriminating carcinoma tissues from the corresponding para‐carcinoma tissues. The CSC‐sustained self‐assembly strategy provides a comprehensive and smart toolbox for organizing various hierarchical DNA nanostructures, which may facilitate more insights for clinical diagnosis and therapeutic assessment.

## Graphical abstract

A catalytic self‐defined circuit (CSC), facilitating hierarchical‐dependent self‐assembly of DNA nanostructure, is developed for robust and reliable discriminating carcinoma tissues and the corresponding para‐carcinoma tissues in clinic samples.

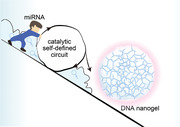

## 1 Introduction

As a basic construct of life, cell is orchestrated by various biomolecular self‐assembly circuitry that further forms complex molecule reaction networks. The cytoskeleton, for example, is an impressive biochemical circuitry network that, under a predefined space and time, controls the mechanical property,^[^ ^1^ ^]^ the transportation of components,^[^ ^2^ ^]^ and the division/motility of host cells.^[^ ^3^, ^4^ ^]^ Mimicking nature's innate ability has enabled the precise fabrication of various artificial biocircuits for regulating cellular behaviors, including proliferation,^[^ ^5^ ^]^ differentiation,^[^ ^6^ ^]^ mobility,^[^ ^7^ ^]^ and defense.^[^ ^8^ ^]^ Extensive efforts have been spent in manipulating the self‐assembled artificial nanostructures for biological exploration,^[^ ^9^, ^10^, ^11^, ^12^ ^]^ drug delivery,^[^ ^13^, ^14^, ^15^, ^16^, ^17^ ^]^ tissue engineering,^[^ ^18^, ^19^ ^]^ and biosensing^[^ ^20^, ^21^, ^22^, ^23^, ^24^ ^]^ applications. However, in practice, the building of dynamic and programmable self‐assembly systems in living systems still represents a major challenge for their complicated design of reaction pathways/reactants that are difficult to reconstitute and reprogram in complex and dynamic physiological environments. Inspired by the cytoskeleton's hierarchical architecture, the concatenated chemical reaction networks have emerged as the powerful paradigm for engineering the self‐assembly systems in vivo.

Nucleic acid nanotechnology offers a new and versatile avenue for mimicking the autonomous behaviors of cytoskeletal filaments by encoding the concatenated reaction circuits into programmable self‐assembly systems. The high predictability and hybridization fidelity of DNA makes it possible to design DNA‐based circuits with emergent properties and functions.^[^ ^25^, ^26^, ^27^, ^28^, ^29^ ^]^ There have been some explorations on the immobilization of circuit elements on DNA‐encoded origami scaffolds,^[^ ^30^, ^31^, ^32^ ^]^ dedicated to the orienting of multi‐step reactions within a confined volume. Such circuitry written into DNA origami forms a benchmark for the construction of synthetic biocircuits. However, the escaped cargo reactants could easily lead to unintentional binding interactions with limited reaction accelerations.^[^ ^33^, ^34^, ^35^, ^36^ ^]^ Furthermore, the colocalization of active sites on intractable DNA origami scaffolds also constrains the modularity and scalability of circuitry design and efficient execution. The self‐defined hierarchically concatenated hybridization circuit represents a promising approach for achieving the sequence‐controlled biosynthesis of DNA nanostructures with user‐defined logic and dynamic behaviors.^[^ ^4^, ^37^, ^38^, ^39^, ^40^, ^41^ ^]^ Particularly, the addressable and cyclic reconfiguration of hierarchically concatenated hybridization circuits allows site‐specific anchoring of biomolecules in situ, which are essential for tuning the input‐output relationships to meet on‐demand functions. Unfortunately, the self‐sustained hierarchically hybridization circuit is still lagging in terms of precision, controllability, and uniformity with increased molecular architecture size.

Herein, we engineered a catalytic self‐defined circuit (CSC) for spatially selective microRNA (miRNA) imaging by the stimulated assembly of hierarchical DNA nanogel. The central idea is to partially sequester palindromic fragments into three hairpin reactants to provide a self‐sustained reaction format, and thus avoiding the external requirement of pre‐assembled building blocks and spurious cross‐interactions. The execution of CSC lies in the re‐configuration of probe structural symmetry to expose the sequestered stem domain upon miRNA stimulation. Once the blocking symmetry sequence is catalytically unfolded by miRNA, the interconnecting catenated DNA reactant is generated to carry out proximal hybridization, thus facilitating the hierarchically concatenated DNA assembly. Meanwhile, the endogenously located miRNA can be re‐generated during the self‐sorting assembly of molecular architecture, thus promoting the on‐site co‐localization of the intracellular analyte with high accuracy. The use of sequestered palindromic fragments brings about a simple, modular design for the minimal CSC with only three hairpins, which promotes high cell‐delivering uniformity and eliminates unintentional cross‐talk between the cumbersome reactants. Profiting from the pre‐sealed palindromic fragments, the CSC system realizes spatially confined proximal hybridization for self‐sustainably assembled DNA products with improved reaction kinetics, exhibiting localization‐intensified cascaded signal amplification. Furthermore, the high molecular weight of self‐sustainably assembled DNA products, as compared with the conventional catalytic hybridization circuit, further promotes the amplified intracellular imaging of analyte in situ. The miRNA‐stimulated hierarchically concatenated self‐assembly strategy could effectively distinguish carcinoma tissues and the corresponding para‐carcinoma tissues, which would represent a versatile avenue for precise manipulation of DNA nanostructures and open new horizons for smart diagnostics and therapeutics.

## 2 Results and Discussion

### 2.1 Principle of the Stimuli‐Responsive Hierarchically Concatenated Circuit for miRNA Assay

**Figure 1:**
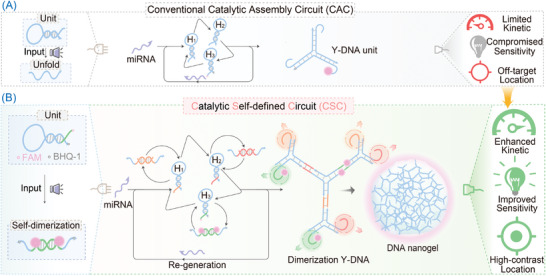
Comparison of the conventional catalytic assembly circuit (CAC) system A) and the hierarchically concatenated catalytic self‐defined circuit (CSC) system B) for miRNA assay. By incorporating pre‐sealed symmetrical fragments into the three preying hairpin reactants, the compact CSC system allows the hierarchical DNA self‐assembly via a miRNA‐powered self‐sorting catalytic hybridization reaction.

The hierarchically concatenated CSC system is implemented via the spatially localized catalytic assembly circuit (CAC) reaction (**Figure** **1**A). In a conventional CAC reaction, each promotor could produce multiple Y‐shaped DNA units by motivating the cross‐opening of three metastable DNA hairpin reactants (Figure S1, Supporting Information), thus contributing to the amplified signal readout. Upon introducing the pre‐sealed symmetrical fragments in the **H_2_** hairpin reactant, the catalytic dimerization circuit (CDC) reaction is realized (Figure S2, Supporting Information). In the CDC system, the unlocked hairpin prey exposes the crucial palindromic domain leading to the co‐localization and dimerization of the CAC reaction, which could in turn impose restrictions on the free diffusion of CAC reactions by the intermolecular interaction to accelerate the rates of DNA assembly. To construct a stimulated assembly of hierarchical DNA nanogel, we deploy pre‐sealed symmetrical fragments into three hairpin prey units to provide a versatile modular framework to simplify the design (Figure 1B; details see Figure S3, Supporting Information). Especially, the pre‐sealed palindromic domain in the stem could eliminate unintentional binding interactions between the spatially inaccessible processing while promoting cascaded proximal hybridization. The design of hairpin **H_1_** with a toehold domain in the loop allows the precise modulation of the energy barriers for self‐assembly, further mitigating leakage to enhance circuit accuracy. Once promotor miR‐155 is introduced to the CSC system, it hybridizes with the loop region of the hairpin **H_1_** to expose the sequestered stem and palindromic region, accompanying the formation of a self‐catenated structure by intermolecular hybridization. The activated **H_1_** opens hairpin **H_2_** by hybridizing with the toehold domain, yielding a **T‐H_1_·H_2_**‐dependent multiplex nanostructure. As a consequence, the opened **H_2_** hybridizes and opens **H_3_** to re‐generate the promoter and produces the Y‐shaped **H_1_·H_2_·H_3_** multiplex. The squeezed‐out promotor can re‐participate in the initial catalytic hybridization reaction, ultimately assembling into the advanced Y‐shaped DNA‐based nanogel by the self‐remodeled assembly principle. This self‐sustainable CSC system streamlined the circuit component with minimal strand complexity for self‐confined assembly of highly branched DNA networks in situ and realized localization‐intensified cascaded signal amplification. Noteworthy, the 5'‐end of **H_3_** is labeled with a FAM fluorophore while the 3'‐end was labeled with a BHQ‐1 quencher (detailed sequence, see Figures S1–S3, Supporting Information). Thus, each miRNA could initiate a “domino” reaction via self‐remodeled catenated circuits, resulting in the efficient fluorescence recovery of FAM for amplified turn‐on fluorescence transduction of the miRNA.

### 2.2 Characterization of the CSC System

**Figure 2:**
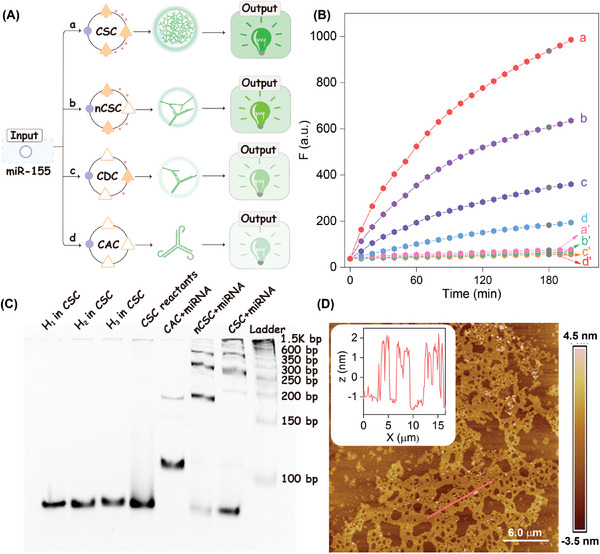
Characterization of the CSC system. A) Comparison of the performance of the different circuit systems: (a) CSC system, (b) nCSC system, (c) CDC system, and (d) conventional CAC system. B) Time‐dependent fluorescence response (at *λ* = 520 nm) of the different circuits outlined in (A) with (a, b, c, and d) or without miRNA promotor (a’, b’ c’ and d’). C) PAGE analysis of the products of the different circuit systems. D) AFM characterization and cross‐section analysis of the CSC‐involved assembly products.

The detailed CSC reaction process was explored by a series of complementary control assays. The miRNA‐catalyzed production of the palindromic regions played a key role in implementing hierarchically concatenated self‐assembly. To validate the advantage of our self‐sustainable CSC system, a non‐auto‐remodeled catalytic circuit (namely, nCSC) was established by replacing the exposed palindromic sequence with an inactive poly thymine sequence in the **H_2_** hairpin reactant for comparison purpose (**Figure** **2**A). As shown in Figure 2B, the mixture of hairpin reactants observed no significant fluorescence variation, indicating that the undesired cross‐talks were effectively precluded by sequestered toehold in the hairpin loop. Upon the presence of the miRNA promotor, a tremendously increased fluorescence response was produced in the intact CSC system, followed by **H_2_**‐substituted nCSC system, while a slightly increased fluorescence signal was observed in the CAC system (for details, see Figure S4, Supporting Information). The variable fluorescence responses were attributed to the autonomously and progressively catalytical hybridization reaction, where the self‐sustained concatenated hybridization reaction of the CSC system resulted in improved reaction kinetics. The optimized reaction temperature of the CSC system was investigated and displayed the best signal‐to‐noise (S/N) ratio at 37 °C (Figure S5, Supporting Information), which is consistent with the physiological temperature. The native polyacrylamide gel electrophoresis (PAGE) assay was further performed to validate the working principle of the CSC system. As expected, no new band was observed for the CSC or nCSC reactants without miRNA (Figure 2C; Figure S6, Supporting Information), while more new bands of remarkably higher molecular weights emerged for miRNA‐stimulated CSC system as compared with miRNA‐initiated nCSC system, ascribing from the self‐sustained concatenated catalysis for accelerating and promoting hierarchically proximal hybridization and generation of dsDNA dendrimers. The morphological characterizations of the CSC‐generated supramolecular copolymers were evaluated by an atomic force microscope (AFM). As exhibited in Figure 2D, the height of CSC‐assembled dsDNA nanocomposites was measured to be ≈1.5 nm, a characteristic height of dsDNA, indicating the feasibility of the interconnected reaction pathway of the CSC system.

**Figure 3:**
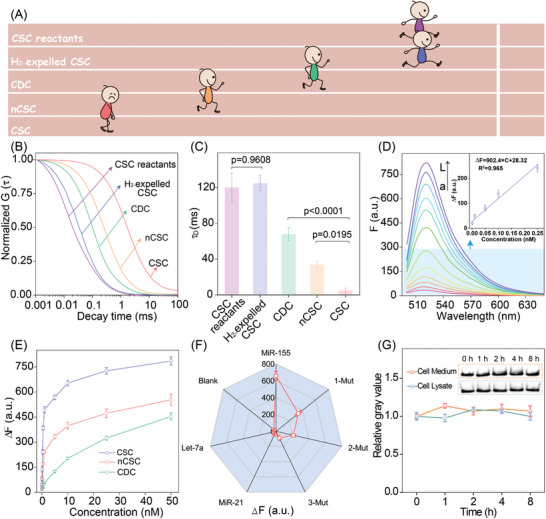
Performance of the CSC amplifier in vitro. A) The scheme of molecular fluctuation for different defective CSC amplifiers and the intact CSC amplifier. B) Normalized FCS curves and C) corresponding diffusion coefficient assay as shown in Figure 3A. Statistical significance is calculated by one‐way ANOVA followed by Tukey's post hoc test. D) Fluorescence spectra of the CSC amplifier in response to miR‐155 promotor with different concentrations: a) 0 pm, b) 1 pm; c) 10 pm; d) 50 pm; e) 100 pm; f) 250 pm; g) 500 pm; h) 1 nm; i) 5 nm; j) 10 nm; k) 25 nm; l) 50 nm. Inset: the calibration curve at a miR‐155 concentration ranging from 1 to 250 pm. E) Fluorescence signal generated by the intact CSC, nCSC, and CDC upon analyzing the same concentration of miR‐155. F) Measured fluorescence response of the CSC amplifier upon analyzing 5 nM miR‐155, mutant miR‐155, interfering miRNAs, and blank (no analyte). G) The PAGE characterization of the CSC reactants in cell culture medium and the cell lysate for different time intervals. Error bars indicated the mean ± SD of three biological replicates.

Subsequently, single‐molecule fluorescence correlation spectroscopy (FCS) was used to investigate the local concentration fluctuations to obtain information about the reaction products (**Figure** **3**A). The CSC DNA reactants (control) observed substantially faster molecular diffusion, while the prey‐substituted nCSC system showed a lower molecular diffusion, yet was still prominently faster than the intact CSC system (Figure 3B). In contrast, the **H_2_**‐expelled defective CSC system observed little effect on the molecule fluctuations, indicating the indispensable integrity of all CSC reactants for the integrated domino hybridization reaction. The CDC system showed 13.2‐fold molecular fluctuation enhancement as compared to the CSC system, and a 2.2‐fold molecular fluctuation reinforcement as compared to the nCSC system (Figure 3C). This remarkably suppressive molecular fluctuation of the CSC system was rationalized by the inherent hierarchically concatenated assembly principle, resulting in enhanced reaction kinetics and generation of dendrimer‐like dsDNA to constrain the molecular diffusion. The successful execution of the hierarchically concatenated CSC system was then explored for sensitive miRNA analysis. Figure 3D exhibited the fluorescence spectra of the CSC amplifier upon 3 h incubation of miR‐155 at various concentrations. A good linear relationship was acquired between the fluorescence intensity change and miR‐155 concentration ranging from 1 to 250 pm with a correlation equation Δ*F* = 902.4 × C + 28.32 (*R* ^2^ = 0.965, C represented the concentration of miR‐155 while Δ*F* represented the corresponding fluorescence intensity change). The detection limit was calculated to be 0.25 pm according to the conventional 3σ/*k* principle (σ is the standard deviation of the 11 background signals and *k* denotes the slope of the calibration curve), which was comparable even better to previous investigations based on nucleic acid circuits (Table S3, Supporting Information). Simultaneously, the sensing performance of the nCSC and CDC systems was also explored to analyze variable concentrations of miR‐155 (Figure 3E). Because the self‐adaptive DNA scaffold was inhibited, the nCSC system observed the detection limit of 42 pm (Figure S7, Supporting Information), and the CDC system acquired the detection limit of 0.18 nM (Figure S8, Supporting Information). The substantially enhanced signal amplification capacity of the CSC amplifier is attributed to the hierarchically concatenated hybridization that underwent localization‐intensified cascaded reaction accelerations. The specificity of this CSC amplifier was verified by the efficient discrimination of these mutant miRNA sequences (Figure 3F). In addition, the robustness of the current CSC system was illustrated by executing the circuitry in cell culture medium, cell lysate, and even 15% serum medium (Figure 3G; Figure S9, Supporting Information). These results significantly demonstrated that the present CSC amplifier could be utilized as a versatile tool for reliable and robust detection of low‐abundance biomolecules.

### 2.3 Intracellular Imaging Performance of the CSC System

**Figure 4:**
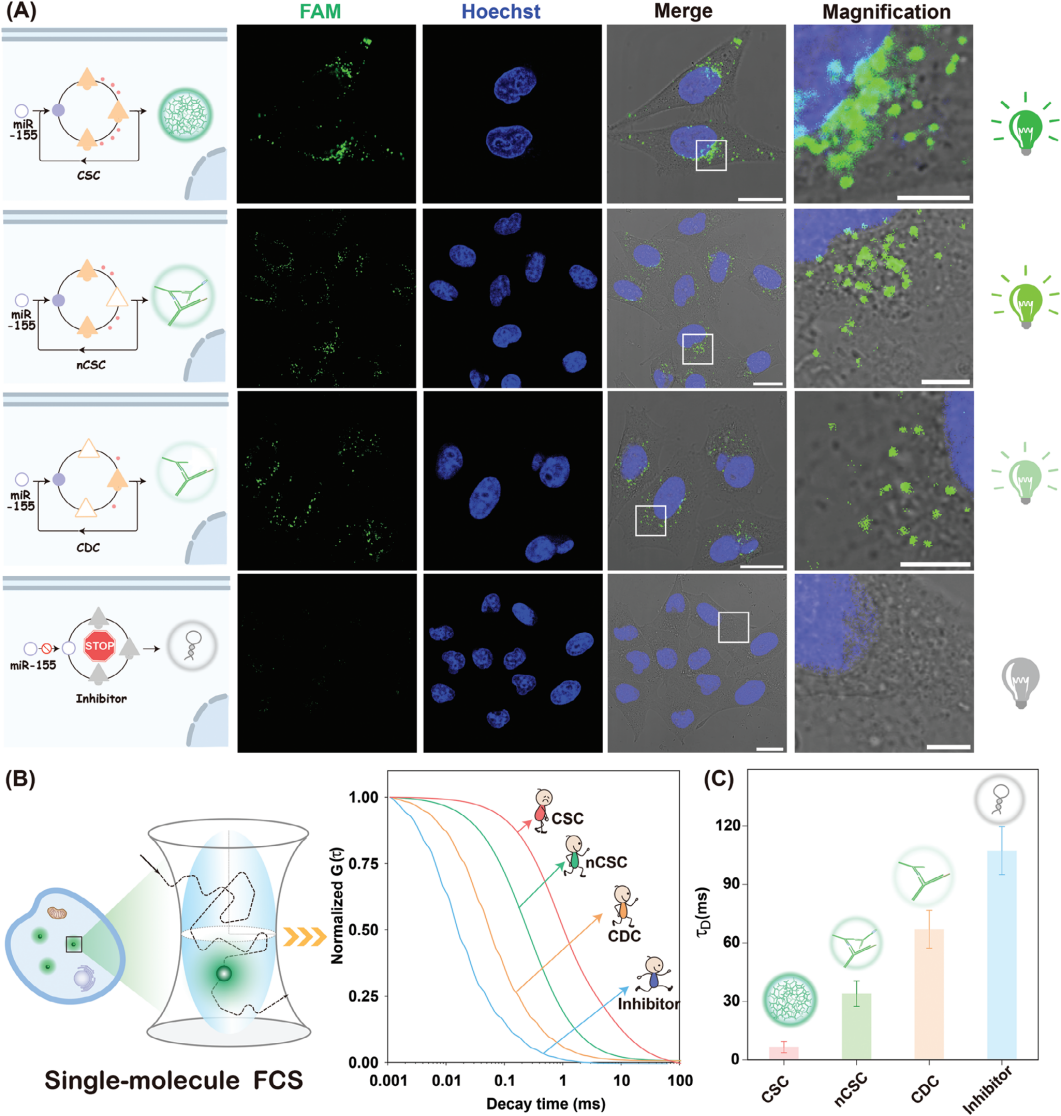
CSC‐sustained miRNA imaging in live MCF‐7 cells. A) CLSM observation and B) Single‐molecule FCS assay of intracellular miR‐155 by intact CSC, nCSC, CDC, and anti‐miR‐155‐pretreated CSC. The scale bars of the merge panel are 20 µm, while the scale bars of the magnification panel are 10 µm. C) Diffusion coefficient assay as described in Figure 4B. Error bars indicated the mean ± SD of five biological replicates.

The satisfactory sensing performance of the CSC amplifier inspired us to explore its characterization in living cells. To improve stability in complex intracellular circumstances, all the CSC probes were partially synthesized with phosphorothioate bonds without affecting their sensing performance (Figure S10, Supporting Information). The modified CSC probes were transfected into living cells using lipofectamine 3000 commercial transfection reagent without remarkable cell cytotoxicity (Figure S11, Supporting Information), indicating the satisfactory biocompatibility of the CSC system that further favors its bioapplication. The cancerous human breast cancer cells (MCF‐7) with relatively high levels of miR‐155 were initially explored to investigate the performance of the CSC‐evoked intracellular amplifier for analyzing miR‐155 expressions. According to the confocal laser scanning microscopy (CLSM) imaging results in Figure S12A (Supporting Information), a gradually increased fluorescence signal was observed with prolonged incubation time and reached a saturation value after 3 h, which correlates well with flow cytometry assay (Figure S12B,C, Supporting Information). Therefore, 3 h was chosen as the optimized incubation duration for realizing an intensified fluorescence response. As shown in **Figure** **4**A, a substantially enhanced cytoplasmic fluorescence signal was observed for the CSC‐imaging system compared to that of the nCSC and CDC systems, indicating the enhanced localization capacity of the CSC‐imaging system inside living cells. The CLSM imaging observation was highly consistent with the results of the flow cytometry assay (Figure S13, Supporting Information). In addition, scarcely any fluorescence readout was observed in anti‐miR‐155 inhibitor pre‐treated MCF‐7 cells, implying that the stimulus‐responsive CSC system could, indeed, proceed in living cells for in situ detection of low‐abundance miR‐155. As compared with the intact CSC imaging amplifier, an enhanced fluorescence signal attenuation was detected in nCSC system‐treated MCF‐7 cells, while the most intense diffusion signal was observed in anti‐miR‐155 inhibitor pretreated cells (Figure 4B). Further quantitative analysis showed that the CDC system achieved 2.1‐ and 10.1‐fold higher fluorescence diffusion than the nCSC system and the intact CSC system. Noteworthy, the introduction of miR‐155 inhibitor resulted in fast molecular diffusion (Figure 4C), which exhibited ≈16.3‐fold faster fluorescence fluctuation, as compared to the CSC system without miR‐155 inhibitor pre‐treatment. The variable molecular diffusive properties originated from the different size distributions of the assembly DNA domino architecture, where the self‐tethered acceleration activation of the CSC system led to the generation of high molecular‐weight hyperbranched dsDNA copolymers with low cellular diffusibility.

**Figure 5:**
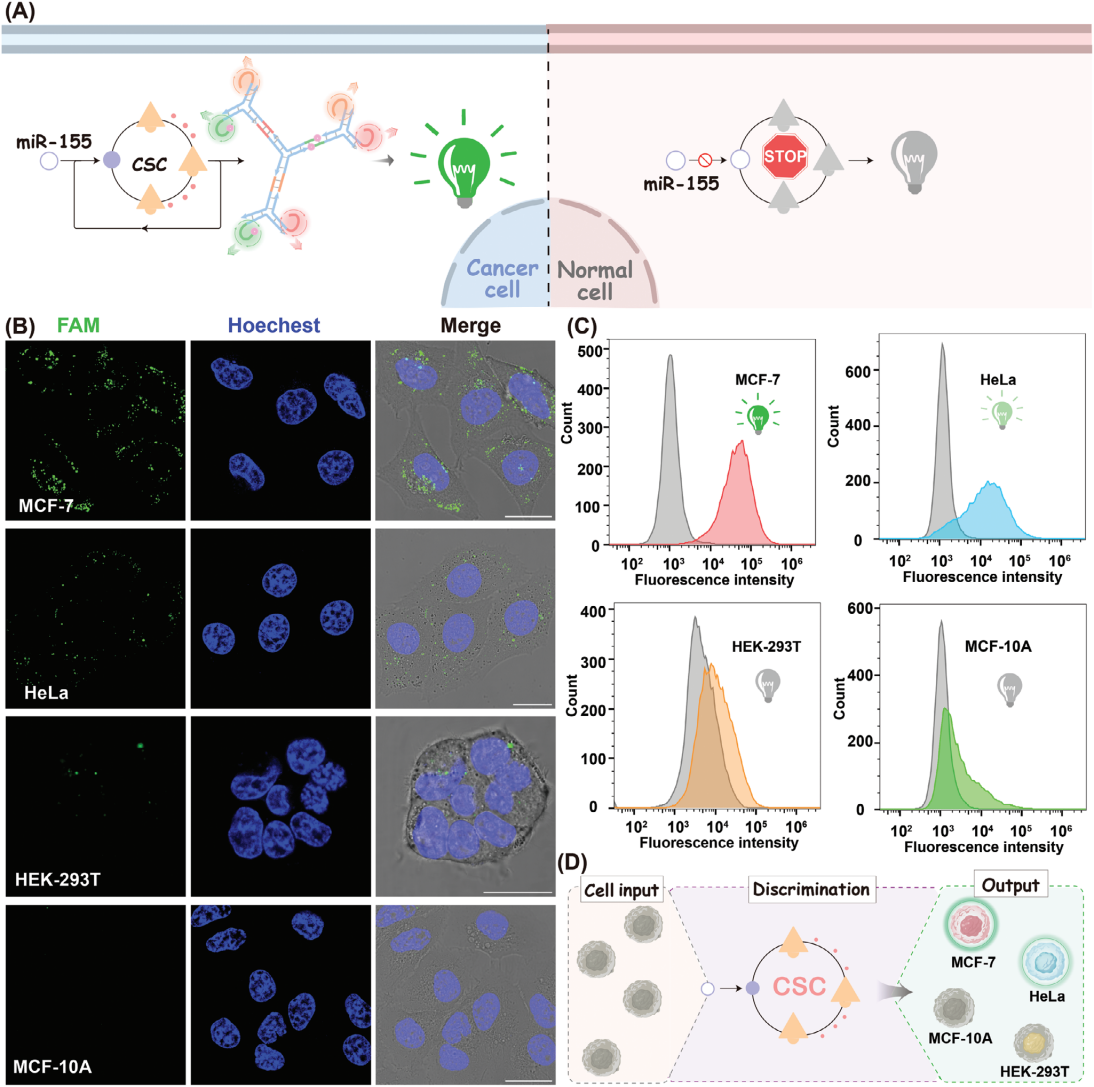
Intracellular miRNA imaging of the CSC amplifier in different cell types. A) Schematic representative of the performance of CSC amplifier in cancerous and normal cells. B) CLSM imaging, and C) Flow cytometry assay of the CSC‐sustained miR‐155 expression profiles in MCF‐7, HeLa, HEK‐293T, and MCF‐10A cells. Scale bars: 20 µm. D) Schematic illustration of the CSC amplifier distinguishing different cell types.

Subsequently, we explored how the CSC amplifier responded to distinct miR‐155 expressions in different cell types, including MCF‐7 with relatively overexpressed miR‐155,^[^ ^42^, ^43^ ^]^ human cervical cancer cells (HeLa) with moderate miR‐155 expression profiles,^[^ ^44^, ^45^ ^]^ human embryonic kidney 293T cells (HEK‐293T) and human normal breast cells (MCF‐10A) with negligible miR‐155 expression levels.^[^ ^46^, ^47^ ^]^ The robustness of the modified DNA probe in these four different cell types was confirmed by flow cytometry analysis (Figure S14, Supporting Information). The cancer cell discrimination ability of the designed CSC system was first explored using CLSM observation (**Figure** **5**A). As shown in Figure 5B, a bright fluorescence spot was observed in MCF‐7 cells, yet a comparatively weaker fluorescence response appeared in HeLa cells. By contrast, almost no detectable fluorescence readout was observed in normal MCF‐10A and HEK‐293T cells, indicating a comparatively higher miR‐155 content in tumor cells, not in normal cells. The quantitative flow cytometry evaluation results were consistent with that of CLSM analysis (Figure 5C; Figure S15, Supporting Information), implying that the fluorescence signal was indeed generated by the miR‐155‐initiated concatenated hybridization reaction and the designed CSC system was able to discriminate different cell types with diverse miR‐155 content. According to the CLSM observation and flow cytometry analysis, the order of miR‐155 content was MCF‐7 > HeLa > HEK‐293T ≈ MCF‐10A, which showed a good agreement with the gold standard quantitative reverse transcription‐PCR (qRT‐PCR) analysis results (Figure S16, Supporting Information). These results indicate the capability of our proposed CSC system to differentiate the cell‐to‐cell variation of miR‐155 expression (Figure 5D), thus, promoting the investigation of miRNA‐related disease pathophysiology.

### 2.4 Sensing Performance of the CSC‐Sustained Amplifier In Vivo

**Figure 6:**
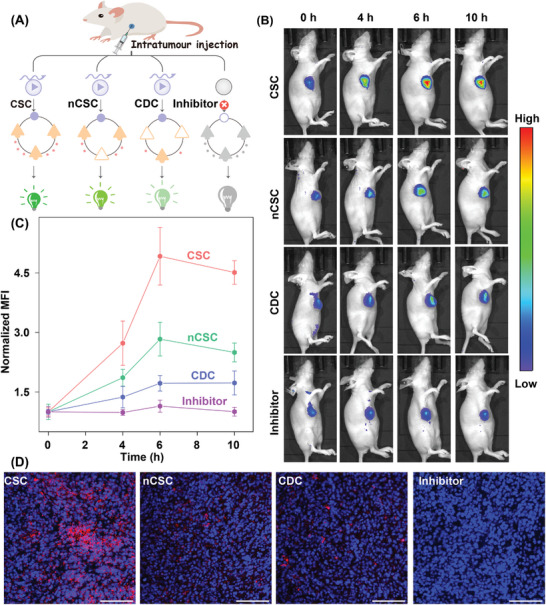
The miRNA imaging performance of the CSC system in orthotopic MCF‐7 tumor‐bearing mice via intratumoral injection. A) Schematic representation of these different amplifiers for miR‐155 imaging in vivo: a) intact CSC system, b) nCSC system, c) CDC system, and d) miR‐155 inhibitor‐pretreated CSC system. B) Whole‐body fluorescence images and C) corresponding normalized mean fluorescence intensity (MFI) at the tumor sites. D) Representative fluorescence images of tumor tissues are described in Figure 6A after 24 post‐injection. Scale bars: 200 µm. Error bars indicated   mean ± SD of three independent assays.

The robustness of the CSC amplifier inspired us to explore its in vivo miR‐155 sensing performance. Here, hairpin **H_3_** was modified with Cy5/quencher pair to enhance light penetration in the tissue. The lipofectamine 3000‐loaded different amplifier was intratumorally injected into orthotopic MCF‐7 tumor‐bearing female BALB/c nude mice with high biocompatibility (**Figure** **6**A; Figure S17, Supporting Information). A distinct fluorescence enhancement was exhibited in the tumor site with the treatment of an intact CSC amplifier, while the **H_2_**‐substituted nCSC system‐treated mice observed relatively weak fluorescence signal (Figure 6B). No noticeable fluorescence change, however, was observed for the miR‐155 inhibitor‐pretreated tumors. Quantitative analysis furtherly displayed that at the tumor site, the CSC amplifier presented a 2.9‐fold higher fluorescence intensity than the CDC system (Figure 6C), 1.8‐fold higher fluorescence readout than the nCSC system, and 4.5‐fold higher fluorescence signal than the miR‐155 inhibitor‐injected CSC system at 6 h post‐injection. These different intratumoral fluorescence response results demonstrated that miR‐155‐responsive self‐adaptively concatenated hybridization reaction could proceed in a complex and challenging in vivo environment for amplified miR‐155 imaging. The substantially improved fluorescence signal of the CSC system was mainly ascribed to the activatable palindromic fragments, facilitating proximal hybridization with localization‐intensified cascaded signal amplification. Fluorescence imaging of tumor sections at 24 h post‐administration observed a trend similar to that of the real‐time tumor imaging assay (Figure 6D). The tread of Cy5 signal in harvested tumors and major organs at 24 h injection was in accordance with in vivo results (Figure S18, Supporting Information). Hardly any hepatic and nephritic dysfunction (Figure S19, Supporting Information) or tissue damage (Figure S20, Supporting Information) was observed in mice treated with the CSC probes, demonstrating its high biocompatibility. Therefore, it is conceivable that this robust CSC amplifier holds great potential for precisely detecting miRNA in vivo.

### 2.5 Performance of the CSC System in Clinical Samples

**Figure 7:**
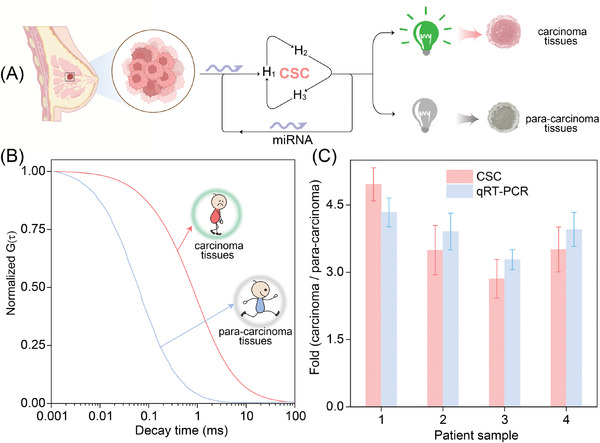
The miRNA sensing performance of the CSC system in clinical samples. A) Schematic representation of the intracellular miR‐155‐lighted CSC system for distinguishing breast carcinoma and corresponding para‐carcinoma tissues. B) Normalized FCS curve assay of miR‐155 content in breast carcinoma tissues and the corresponding para‐carcinoma tissues. C) Comparison of miR‐155 expression profiles by the CSC system and conventional qRT‐PCR in breast carcinoma tissues and the corresponding para‐carcinoma tissues from 4 patients. Error bars indicated mean ± SD of five biological replicates.

For clinical evaluation of the CSC system, the designed CSC reactants were then introduced to probe the miR‐155 content in carcinoma tissues and the corresponding para‐carcinoma tissues (**Figure** **7**A). As expected, a substantially enhanced signal attenuation was observed in para‐carcinoma tissues as compared to that in carcinoma tissues (Figure 7B), indicating that the CSC amplifier is, indeed, suitable for detection of less‐abundantly expressed miRNA from different tissues of carcinoma patients. As compared to the para‐carcinoma tissues, the expression profile of miR‐155 in 4 of the breast cancer patients was relatively over‐expressed in carcinoma tissues (Figure 7C), which is compelling agreement with the qRT‐PCR results, indicating the miR‐155‐specific tissue‐distinguishing reliability of our CSC amplifier from carcinoma patients.

## 3 Conclusions

In conclusion, we rationally demonstrated the construction of hierarchically concatenated hybridization circuit for minimal cytoskeleton mimics through miRNA‐responsive self‐sorting catalytic DNA hybridization reaction. The compact CSC system simplified the elaborate components of conventional catalytic assembly circuits into only three hairpin reactants, resulting in high cell‐delivering uniformity and minimal signal leakage. The partially sequestered symmetry fragments in the CSC system guaranteed self‐adapted biocatalytic cascades and high reaction kinetics. The high‐molecular‐weight of hyperbranched DNA domino nanoproducts, as compared to the traditional catalytic hybridization monomers, facilitates the on‐site co‐localization of intracellular analyte with high accuracy, which can help a comprehensive understanding of intracellular metabolism process. Benefiting from the localization‐intensified signal amplification, this CSC system not only realized robust miRNA imaging in tumor tissues but also implemented the discrimination of different tissues with varied miRNA expression profiles from breast carcinoma patients. Considering the programmability of the DNA, our developed compact CSC system paves a new avenue for developing hierarchical DNA nanostructures, which expands the application of DNA‐aided nanotechnology for biomedical engineering and nanofabrication.

## 4 Experimental Section

### Materials

The HPLC‐purified oligonucleotides were synthesized by Sangon Biotech Co., Ltd. (Shanghai, China), and the detailed sequences were listed in Table S1 (Supporting Information). Lipofectamine 3000 transfection Reagent, Dulbecco's modified Eagle's medium (DMEM), Opti‐MEM, Fetal Bovine Serum (FBS), trypsin, penicillin‐streptomycin, Hoechst 33342, and Dulbecco's phosphate buffered saline (PBS) were both purchased from Thermo Fisher. Human breast cancer cells (MCF‐7), human cervical cancer cells (HeLa), human embryonic kidney 293T cells (HEK 293T cells), and human normal breast epithelial cells (MCF‐10A) were obtained from Shanghai Institutes for Biological Sciences (SIBS). GelRed was obtained from Invitrogen (Carlsbad, CA, USA). All of these chemicals were of analytical grade and used without further purification.

### Fluorescence Assays

Fluorescence experiments were performed in reaction buffer (10 mm HEPES, 1 m NaCl, 50 mm MgCl_2_, pH 7.2). For evaluation of the assembly performance of different circuit systems, the miR‐155 initiator was incubated with their respective DNA mixtures (200 nm each) to initiate the self‐assembly process at 37 °C. The time‐dependent fluorescence responses and fluorescence intensities were conducted by a Cary Eclipse spectrometer (Varian Inc) with *λ*ex = 490 nm. The fluorescence intensity change (Δ*F*) was calculated by the formula “Δ*F* = *F* − *F*_0_” (F represented the fluorescence intensity at the end of the measurement, while *F*_0_ refers to the initial fluorescence intensity).

### Native Polyacrylamide Gel Electrophoresis

The DNA copolymers of the different circuit systems were characterized by 7% native polyacrylamide gel electrophoresis. 20 nM miR‐155 was incubated with 200 nM of their corresponding hairpin mixtures in reaction buffer (10 mm HEPES, 1 m NaCl, 50 mm MgCl_2_, pH 7.2) for 3 h at 37 °C. Then, 10 µL of resultant mixtures were mixed with 2 µL of 6×loading buffer and loaded into the notches of freshly prepared. Electrophoresis was executed at 100 V in 1× TBE buffer (89 mm Tris, 89 mm boric acid, 2.0 mm EDTA, pH 8.3) for 180 min. After staining with diluted GelRed, the gel was imaged by a FluoChem FC3 (Protein Simple, USA) imaging system under 365 nm UV irradiation.

### FCS Measurements

All the FCS measurements were executed in a Leica TCS SP8 confocal laser scanning microscope with a 63 × 1.2 NA water immersion objective. Herein, the hairpin H_3_ was functionalized with Atto 488 at the 3' end. The prepared DNA samples (50 nM) were diluted and deposited in glass‐bottom culture dishes and excited by a 488 nm laser line accompanying emission ranging from 495 to 525 nm. The obtained FCS results were analyzed by using the Symphotime 64 software.

### Sensing Performance in Live Cells

MCF‐7, HeLa, and HEK 293T cells were cultivated in DMEM supplemented with 10% (v/v) FBS and antibiotics (100 U mL^−1^ penicillin and 100 U mL^−1^ streptomycin), while MCF‐10A cells were cultivated in a complete growth medium (Procell Life Science & Technology Co., Ltd.). All of these above cells were grown on a T25 cell culture flask in a humidified atmosphere containing 5% CO2 at 37 °C. After being digested with trypsin, cells were seeded into glass‐bottom cell culture dishes and incubated for 24 h until adherent. The miR‐155‐initiated CSC DNA mixtures (0.2 nmol) were prepared in Opti‐MEM (150 µL) and then mixed with 5 µL lipofectamine 3000 in 150 µL opti‐MEM for 10 min. Subsequently, the plated cells were incubated with the above solutions for 3 h. Then, the cells were washed three times with PBS and stained with Hoechst 33342 for 10 min at 37 °C. After washing three times with PBS, the cells were observed and imaged by a Leica TCS SP8 laser scanning confocal microscope with the Leica Application Suite Advanced Fluorescence (LAS‐AF) software system. To downregulate miR‐155 content, the anti‐miR‐155 inhibitor (0.1 nmol) was prepared in Opti‐MEM (100 µL), and was then mixed with lipofectamine 3000 (3 µL) dispersed in Opti‐MEM (100 µL) for 5 min. Subsequently, the prepared Opti‐MEM transfection mixture was introduced into the plated MCF‐7 cells for 3 h at 37 °C, followed by transfection and incubation with the CSC system for 3 h. All images were acquired at 63 × 1.4 object with oil. The green channel of FAM fluorescence was acquired using an external 488 nm excitation and the blue channel of the Hoechst 33342 was obtained using 405 nm excitation. Meanwhile, cells were trypsinized and suspended in 300 µL cold PBS and analyzed by flow cytometry (Beckman Coulter, USA.).

### The Biosafety Evaluation

First, the cytotoxicity on MCF‐7 and MCF‐10A cells were evaluated by 3‐(4,5‐dimethylthiazol‐2‐yl)‐2,5‐diphenyltetrazolium bromide (MTT) assay. MCF‐7 and MCF‐10A cells were seeded into a 96‐well plate at the density of 1 × 10^4^ cells per well and incubated for 24 h until adherent. Then, different concentrations of the CSC reactants were transfected into these bove cells, respectively, and incubated for 24 h at 37 °C. After incubation, cells were washed with cold PBS three times and 100 µL of MTT solution (1 mg mL^−1^ in PBS) was added to each well and incubated for another 4 h. Subsequently, the supernatant was carefully removed and the formazan crystals were dissolved in 150 µL DMSO with gently shaking for 15 min. The absorbance at the wavelength of 490 nm was measured with a microplate reader (Thermo Scientific). The cell viability was calculated as:

(1) $$\mathrm{Cell}\;\mathrm{viability}=\left(OD_{\mathrm{treated}}-OD_{\mathrm{blank}}\right)/\left(OD_{\mathrm{control}}-OD_{\mathrm{blank}}\right)$$

For the hemocompatibility assay, fresh blood was obtained from BALB/c mice. RBCs were acquired by centrifuging at 1000 g at 4 °C for 5 min. The obtained RBCs were then washed several times with cold PBS until the supernatant was colorless. Subsequently, different concentrations of the lipofectamine 3000‐packaged CSC reactants were incubated with 2% red blood cell (RBC) solution (500 µL) for 4 h at 37 °C. PBS and deionized water (DI) were used as negative and positive controls, respectively. After incubation, the above solution was centrifuged at 1200 g for 5 min at 4 °C and the supernatant was used for absorbance measurement at 570 nm with a microplate reader. The hemolysis percentage was calculated as:

(2) $$\mathrm{Hemolysis}\;\left(\%\right)=\left(A_{\mathrm{sample}}-A_{\mathrm{negtive}}\right)/\left(A_{\mathrm{positive}}-A_{\mathrm{negtive}}\right)\;\times \;100\%$$

Meanwhile, BALB/c mice were injected intravenously with the lipofectamine 3000‐loaded CSC system. After 24 h, the mice were sacrificed and the blood was collected for hematology and blood biochemistry analysis. In addition, the main organs were collected for hematoxylin and eosin (H&E) staining assay.

### The In Vivo Imaging in Orthotopic MCF‐7 Tumor‐Bearing Nude Mice Model

All animal experimental procedures were approved by the Institutional Animal Care and Use Committee of the Animal Experiment Center of Wuhan University (No. S07918050D). Four‐ to six‐week‐old female BALB/c nude mice were purchased from Charles River Company and raised in a specific pathogen‐free grade laboratory according to guidelines for laboratory animals established by the Wuhan University Center for Animal Experiment/A3‐Lab. For establishment of orthotopic MCF‐7 tumor‐bearing female BALB/c nude mice model, MCF‐7 cells (2.0 × 10^6^) mixed with an equal volume of Matrigel (Corning), were orthotopically injected into the third mammary fat pad of female nude mice. When the tumors were grown to ≈100 mm^3^, the tumor‐bearing mice were intratumorally administrated with lipofectamine 3000‐transfected CSC system (5 nmol kg^−1^ each), lipofectamine 3000‐transfected nCSC system (5 nmol kg^−1^ each), lipofectamine 3000‐transfected CDC system (5 nmol kg^−1^ each), and miR‐155 inhibitor pretreated lipofectamine 3000‐transfected CSC system (5 nmol kg^−1^ each). The whole‐body fluorescence images were acquired by an IVIS spectrum system at designed time intervals. After 24 h post‐administration, the mice were euthanized and the major organs and tumors were collected for bioimaging analysis.

### qRT‐PCR Assay in Tissue Samples

Zhongnan Hospital of Wuhan University collected the clinic samples. All the experimental procedures were performed in accordance with the ethical standard as formulated in the Helsinki Declaration and approved by the ethics committee at Zhongnan Hospital of Wuhan University (No. 00270444). Trizol (Invitrogen) was to extract total RNAs from the human participants of the present study and quantified by NanoDrop Spectrophotometer. The Mir‐X miRNA First‐Strand Synthesis Kit (Takara, Dalian) was used to prepare the cDNA. Meanwhile, U6 was used as an endogenous normalization control supplied with the kit.

## Conflict of Interest

The authors declare no conflict of interest.

## Supporting information

Supporting Information

## Data Availability

The data that support the findings of this study are available in the supplementary material of this article.
