# Supplementary material for: Scaling up of a Self‐Confined Catalytic Hybridization Circuit for Robust microRNA Imaging
Source: Adv Sci (Weinh). 2024 Apr 13;11(22):2400517. doi: 10.1002/advs.202400517 (PMC11165520; doi:10.1002/advs.202400517)
Supplement: Supplementary file 1 — Supporting Information [file ADVS-11-2400517-s001.pdf]

ADVANCED SCIENCE
Open Access

Supporting Information

for *Adv. Sci.*, DOI 10.1002/advs.202400517

Scaling up of a Self-Confined Catalytic Hybridization Circuit for Robust microRNA Imaging

Xue Gong, Ruomeng Li, Jiajia Zhang, Pu Zhang, Zhongwei Jiang, Lianzhe Hu, Xiaoqing Liu, Yi Wang and Fuan Wang**

Scaling up of a self-confined catalytic hybridization circuit for robust microRNA imaging

Xue Gong [a, b, #], Ruomeng Li [a#], Jiajia Zhang [b], Pu Zhang [c], Zhongwei Jiang [b], Lianzhe Hu [b], Xiaoqing Liu [a], Yi Wang [b], Fuan Wang [a]**

[a] Department of Gastroenterology, Zhongnan Hospital of Wuhan University, College of Chemistry and Molecular Sciences, Wuhan University, Wuhan 430072, China

[b] Engineering Research Center for Biotechnology of Active Substances (Ministry of Education), Chongqing Key Laboratory of Green Catalysis Materials and Technology, College of Chemistry, Chongqing Normal University, Chongqing 401331, P. R. China

[c] College of Pharmacy, Chongqing Medical University, Chongqing 400016, P. R. China

[#] These authors contributed equally to this work.

[*] To whom correspondence should be addressed. E-mail:

Yi Wang, E-mail: ywang@cqnu.edu.cn

Fuan Wang, E-mail: fuanwang@whu.edu.cn

Table of Contents

Table S1. Sequences of the oligonucleotides in present study S2

Table S2. Sequences of CSC amplifier for bioimaging S3

Figure S1. Schematic representation of the CAC system. S4

Figure S2. Schematic representation of the CDC system. S5

Figure S3. Schematic representation of the CSC system. S6

Figure S4. Fluorescence response of different circuit systems. S7

Figure S5. The optimized incubation temperature. S8

Figure S6. The PAGE characterization of different circuit systems. S9

Figure S7. Performance of the nCSC for miR-155 analysis. S10

Figure S8. Performance of the CDC for miR-155 analysis. S11

Figure S9. Stability of the CSC system in diluted serum samples. S12

Figure S10. The sensing performance of the phosphorothioated CSC system. S13

Figure S11. Cytotoxicity evaluation of the CSC system. S14

Figure S12. Time-dependent sensing performance of the CSC amplifier in live cells......... S15

Figure S13. The intracellular fluorescence signal of the miRNA-initiated CSC system...... S16

Figure S14. The robustness of the modified DNA probe in different cell types S18

Figure S15. The miRNA-specific distinction of different cells S19

Figure S16. The qRT-PCR analysis of miR-155 expression in different cell types S20

Figure S17. The biocompatibility evaluation of the CSC system.. .... S21

Figure S18. *Ex vivo* fluorescence imaging.. .... S22

Figure S19. Hematology and biochemical analyses of the CSC system.. .... S23

Figure S20. Representative H&E-stained images of the main organs.. .... S24

Table S3. Comparison of different nucleic acid sensing methods S25

Table S1. Sequences of the oligonucleotides for miRNA stimulus-responsive system

Name	Sequence (5'-3')
**miR-155**	UUAAU GCUAA UCGUG AUAGG GGU
**Let-7a**	UGA GGU AGU AGG UUG UAU AGU U
**1-Mut**	UUAAU GCUAA U***G***GUG AUAGG GGU
**2-Mut**	UUAAU ***A***CUAA U***G***GUG AUAGG GGU
**3-Mut**	UUAAU ***A***CUAA U***G***GUG AUA**A**G GGU

The bold italic nucleotides of mutant (Mut) indicate the mismatched sequence

Table S2.The oligonucleotide sequences of CSC amplifier for bioimaging

**Name**	**Sequence (5'-3')**
**H1**	G*G*T*G*T*CGACACC TTAAT GCTAA TCGTC CATCC ACCCC TATCA CGATT AGCAT TAA*G*G* T*G*
**H2**	G*G*T*G*G*GCCCACC TTAAT GCTAA TCGTG GTGAG ACGGG ATGGA CGATT AGCAT TAA*G*G* T*G*
**H3**	FAM-G*G*C*G*A*CGTCGCC TTAAT GCTAA TCGTG ATAGG GGTCG TCTCA CCACG ATTAG CATTA A*G*G*C*G*-BHQ-1
**H3***	Cy5-G*G*C*G*A*CGTCGCC TTAAT GCTAA TCGTG ATAGG GGTCG TCTCA CCACG ATTAG CATTA A*G*G*C*G*-BHQ-2
**miR-155 inhibitor**	mA*mC*mC*mCmCmUmAmUmCmAmCmGmAmUmUmAmGmCmA mUmU*mA*mA*

* = Phosphorothioate Bonds

mN = 2'-*O*-Me RNA base

Schematic illustration of the CAC system

The stimulus-responsive CAC system for the assembly of the Y-shaped DNA units was schematically illustrated in **Figure S1**. The region c of miRNA promotor is complementary to the loop c* of $\mathbf{H_1}$ and could thus open $\mathbf{H_1}$ through toehold-mediated strand displacement (**Figure S1A**). The as-exposed domain a-b-d of $\mathbf{H_1}$ immediately hybridizes with domain a*-b*-d* of $\mathbf{H_2}$ for opening $\mathbf{H_2}$. The exposed domain e-b-a of $\mathbf{H_2}$ hybridizes with the sequence e*-b*-a* of $\mathbf{H_3}$ and opens $\mathbf{H_3}$, which leads to the regeneration of the promotor and simultaneous assembly of a Y-shaped DNA units. The opened $\mathbf{H_3}$ results in the efficient separation of the fluorophore/quencher pair (FAM/BHQ-1), thus substantially restoring the fluorescence of FAM. The DNA sequence of the proposed CAC system is shown in **Figure S1B**.

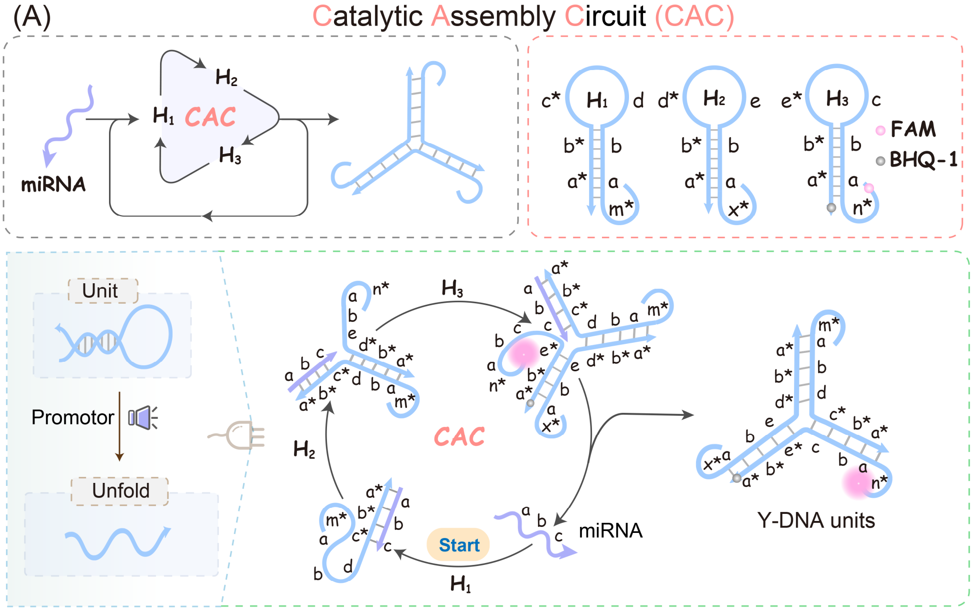

(B) The DNA sequence of the CAC system

H_1	5’-TTTTTTTTCACC TTAAT GCTAA TCGTC CATCC ACCCC TATCA CGATT AGCAT TAAGG TG-3’
H_2	5’-TTTTTTTTCACC TTAAT GCTAA TCGTG GTGAG ACGGG ATGGA CGATT AGCAT TAAGG TG-3’
H_3	5’-FAM-TTTTTTTTCGCC TTAAT GCTAA TCGTG ATAGG GGTCG TCTCA CCACG ATTAG C ATTAA GGCG-BQH-1-3’

Figure S1. **Construction of the CAC system.** (A) The detailed reaction process of the CAC system. (B) The DNA sequence of the CAC system.

Schematic illustration of the CDC amplifier

The miRNA-responsive CDC system for the polymerization of Y-DNA units is shown in **Figure S2**. The region c of miRNA is complementary to the toehold c* of $\mathbf{H_1}$ and thus opens $\mathbf{H_1}$ through toehold-mediated strand displacement (**Figure S2A**). The opened $\mathbf{H_1}$ then hybridizes and opens $\mathbf{H_2}$, which leads to the unlocked palindromic domain (x) and simultaneous formation of symmetry structural. The newly exposed region e-b-a of $\mathbf{H_2}$ could subsequently hybridize with the sequence e*-b*-a* of $\mathbf{H_3}$ to dimerization Y-shaped DNA units and regenerate miRNA. The opened $\mathbf{H_3}$ resulted in the efficient fluorescence recovery of FAM and generated an amplified fluorescence readout. The liberated miRNA could hybridize with another $\mathbf{H_1}$ to trigger the next CDC cycle. The DNA sequence of the proposed CDC system is shown in **Figure S2B**.

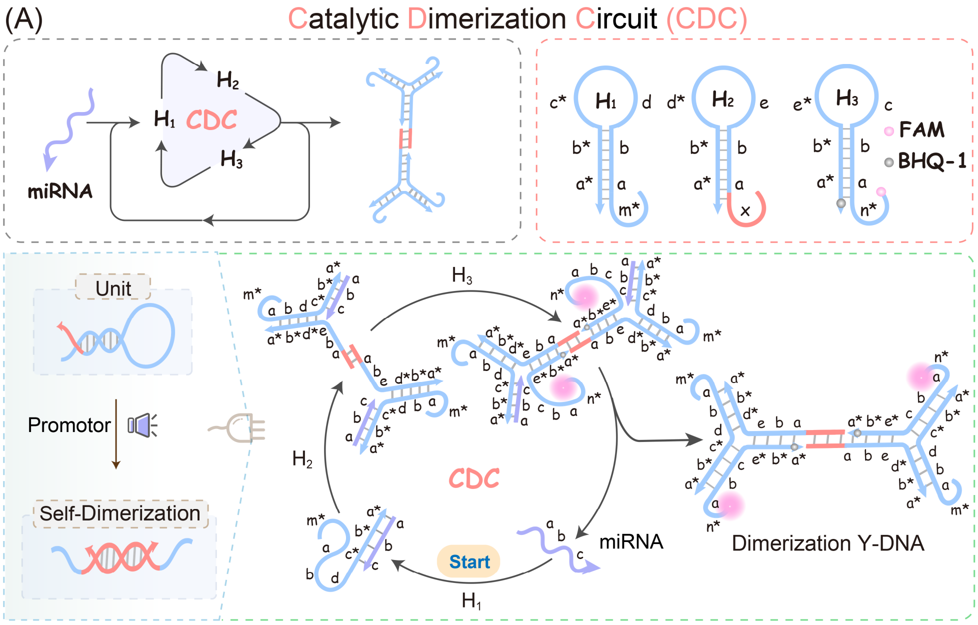

(B) **The DNA sequence of the CDC system**

H_1	5'-TTTTTTTTCACC TTAAT GCTAA TCGTC CATCC ACCCC TATCA CGATT AGCAT TAAGG TG-3'
H_2	5'-GGTGGGCCCACC TTAAT GCTAA TCGTG GTGAG ACGGG ATGGA CGATT AGCAT TAAGG TG-3'
H_3	5'-FAM-TTTTTTTTCGCC TTAAT GCTAA TCGTG ATAGG GGTCG TCTCA CCACG ATTAG C ATTAA GGCG-BHQ-1-3'

Figure S2. **Construction of the CDC amplifier.** (A) The detailed reaction process of the CDC system. (B) The DNA sequence of the CDC system.

Schematic illustration of the CSC system

Profiting from the pre-blocked palindromic fragments in the stem domain, this compact CSC system allows reactant-to-template-mediated assembly. In the presence of the promotor (miRNA), the interconnecting catenated DNA reactant was generated to carry out proximal hybridization (**Figure S3A**), facilitating hierarchically concatenated DNA assembly. The DNA sequence of the CSC system is shown in **Figure S3B**.

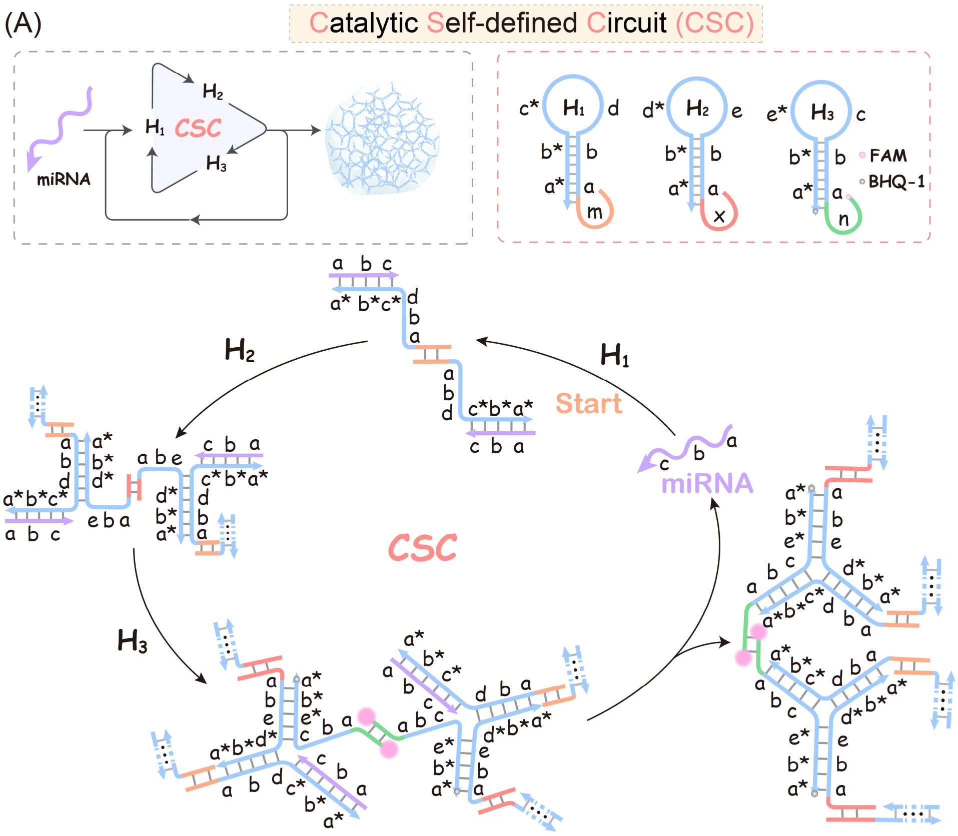

(B) The DNA sequence of the CSC system

H_1	5'-GGTGTCGACACC TTAAT GCTAA TCGTC CATCC ACCCC TATCA CGATT AGCAT TAAGG TG-3'
H_2	5'-GGTGGGCCCACC TTAAT GCTAA TCGTG GTGAG ACGGG ATGGA CGATT AGCAT TAAGG TG-3'
H_3	5'-FAM-GGCGACGTCGCC TTAAT GCTAA TCGTG ATAGG GGTCG TCTCA CCACG ATTAG C ATTAA GGCG-BHQ-1-3'

Figure S3. Illustration of the working mechanism of the miR-155-responsive CSC-involved sensing platform.

The essential role of the pre-blocked symmetrical fragments

The indispensable role of the pre-blocked palindromic fragments in the CSC amplifier was extensively explored by the fluorescence assay. A dramatically enhanced fluorescence signal was observed for the miR-155-initiated intact CSC system (**Figure S4A**), while a relatively low fluorescence readout was revealed in the same miR-155-trigged nCSC system (**Figure S4B**). The enhanced fluorescence response of the CSC system suggested that the pre-blocked palindromic fragment is essential for the promoted templated assembly and the localization-intensified signal amplification.

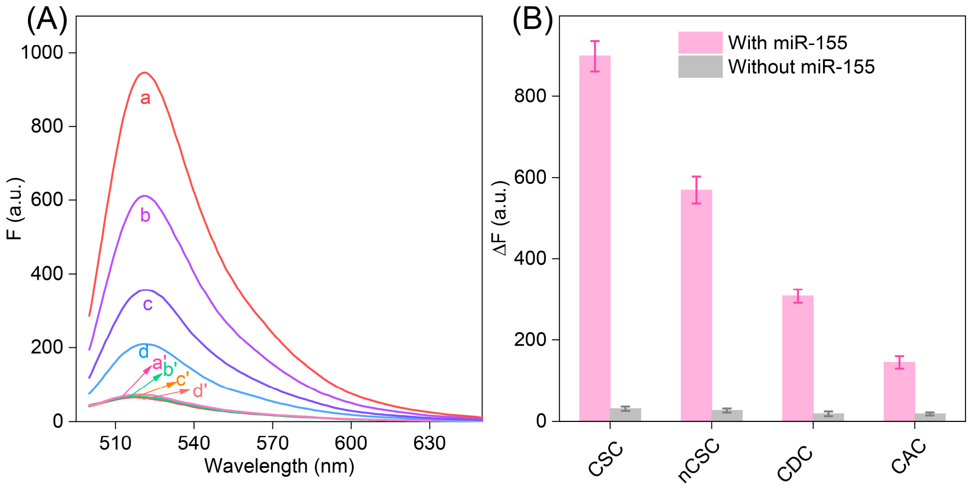

Figure S4. **Fluorescence response of different circuit systems.** (A) Fluorescence spectra and (B) the corresponding statistical histogram of the fluorescence intensity (λ=520 nm) from A. (a') the CSC, (a) the CSC + miR-155, (b') nCSC, (b) nCSC + miR-155, (c') CDC, (c) CDC + miR-155, (d') conventional CAC, (d) conventional CAC + miR-155. Error bars indicate the mean ± SD of three biological replicates.

The optimized incubation temperature

The reaction temperature is an essential factor that affects the thermodynamics of the association/disassociation process of the DNA duplex, thus affecting the sensing performance of the CSC amplifier. The optimized reaction temperature of the CSC system was investigated and displayed the best signal-to-noise (S/N) ratio at 37 °C (**Figure S5**). Therefore, the optimum incubation temperature was chosen as 37 °C for the following experiments.

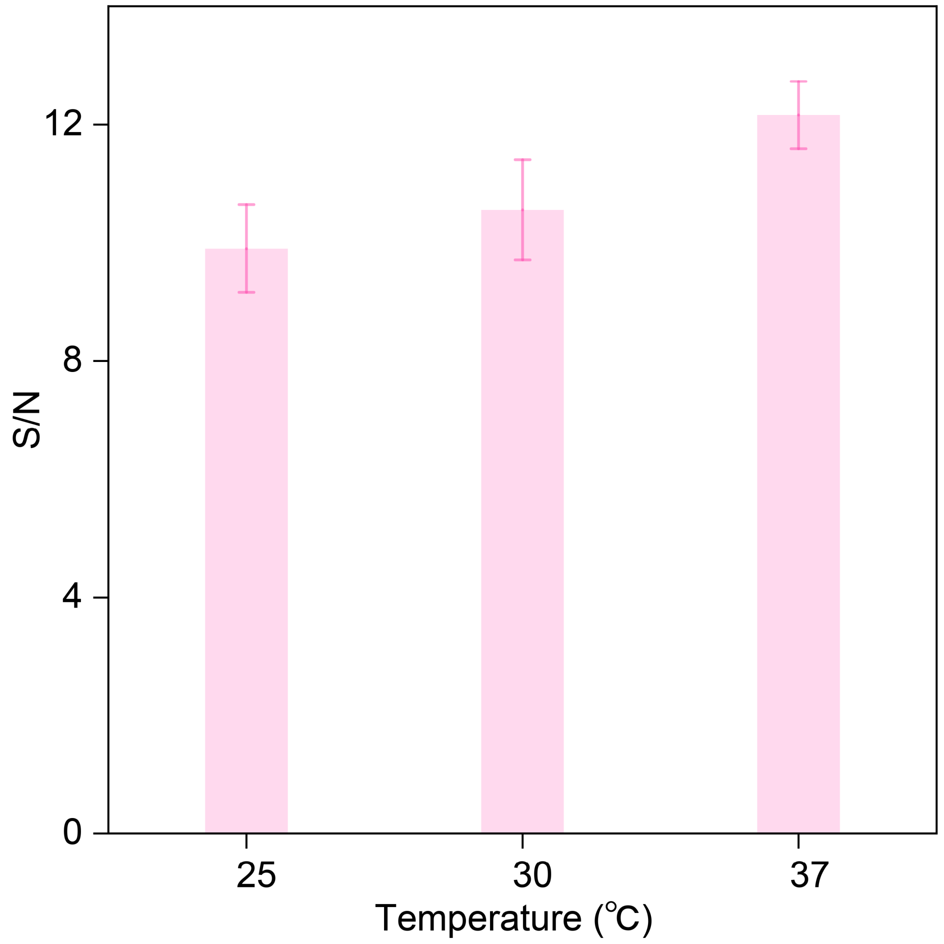

Figure S5. The signal-to-noise ratio (S/N) of the CSC system at different temperatures. The concentration of CSC probe was 200 nM, while the concentration of miR-155 is 0.5 nM. Error bars indicate the mean ± SD of three biological replicates.

Characterization of the miR-155-responsive CSC system

To demonstrate the hierarchically concatenated hybridization process, the native PAGE experiment was performed. As depicted in **Figure S6**, the reaction mixtures observed almost no hybridization product in the absence of miR-155 (lanes 1 to 4), suggesting robustness of our proposed circuit system without apparent signal leakage. By contrast, the introduction of miR-155 into the DNA mixtures resulted in the generation of various new bands with a retarded mobility (lanes 5 to 8) ascribed to the miR-155-responsive assembly performance. As compared to the miRNA-initiated nCSC system, the miRNA-stimulated CSC system observed more new bands of remarkably higher molecular weights, indicating the self-sustained concatenated catalysis for accelerating and promoting hierarchically proximal hybridization and generation of dsDNA dendrimers.

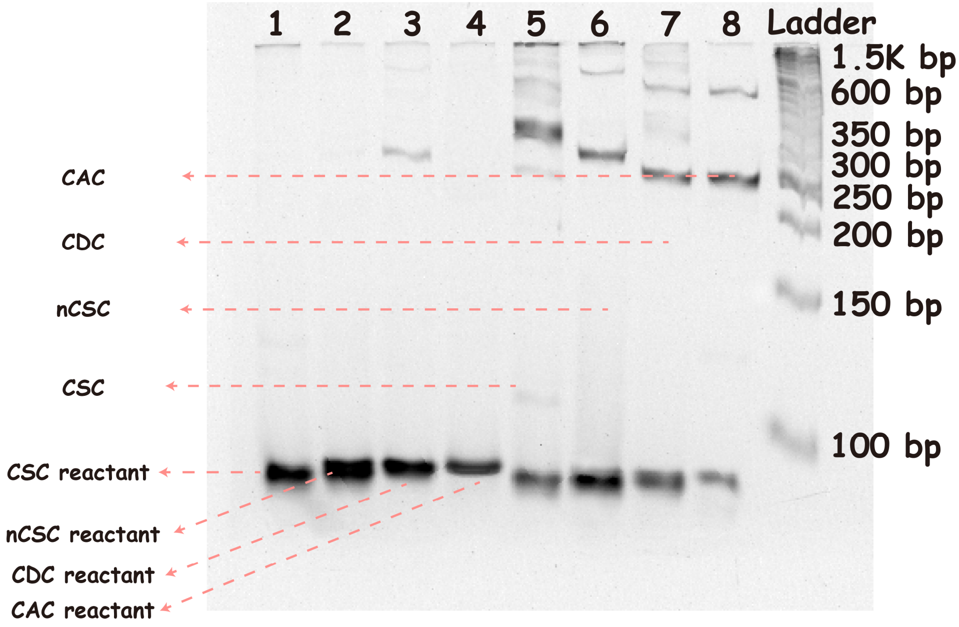

Figure S6. The native PAGE characterization of different circuit systems. The concentration of DNA reactants is 200 nM, while the concentration of miR-155 is 10 nM.

Performance of the nCSC system

Figure S7A shows the real-time fluorescence monitoring of the H_2-substituted nCSC system upon analyzing different concentrations of the miR-155. As displayed in **Figure S7B**, a gradually enhanced fluorescence intensity was observed in response to continuously increased concentrations of the miR-155 ranging from 0.1 to 50 nM. A linear relationship was observed between the fluorescence intensity change (λ=520 nm) and miR-155 with concentrations ranging from 0.1 to 0.5 nM. The regression equation was achieved to be $\Delta F=257.6\times C + 9.998$ with a correlation coefficient of $R^2=0.980$. The detection limit was calculated to be 42 pM according to the $3\sigma/k$ method (σ is the standard deviation of the 11 background signal, while k refers to the slope of fluorescence intensity *vs* the miRNA concentration). Therefore, the intact CSC amplifier reveals better sensing performance than the H_2-substituted nCSC amplifier, confirming the high signal amplification capacity of the localization-accelerated reaction profiles.

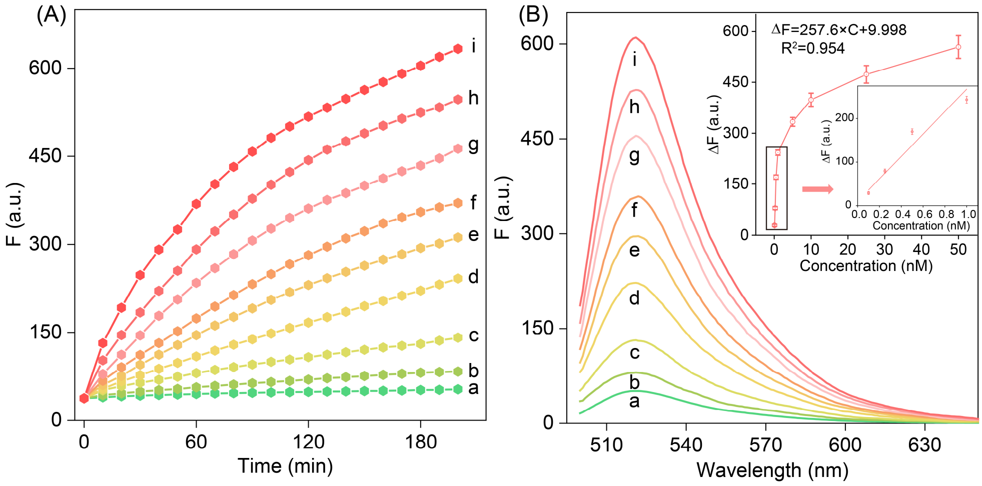

Figure S7. Performance of the H_2-mutant nCSC for miR-155 analysis. (B) Time-dependent fluorescence changes of the nCSC amplifier incubated with different concentrations of promotor miR-155 : (a) 0 nM, (b) 0.1 nM, (c) 0.25 nM, (d) 0.5 nM, (e) 1 nM, (f) 5 nM, (g) 10 nM, (h) 25 nM, and (i) 50 nM. (B) The corresponding fluorescence spectra as shown in S7A at 180 min. Inset: calibration curve of fluorescence intensity change (λ=520 nm) at a miR-155 concentration ranging from 0.1 to 1 nM. Error bars indicate the mean ± SD of three biological replicates.

Performance of the CDC system

Meanwhile, the CDC system was established to demonstrate signal transduction. The fluorescence intensity (λ=520 nm) of our CDC system enhanced with increasing concentration of the miR-155 (**Figure S8A**). A good linear relationship was acquired between the fluorescence intensity change and miR-155 concentration ranging from 0.5 to 10 nM with a correlation equation $\Delta F=18.15\times C+27.56$ (R^2=0.980, C represented the concentration of miR-155 while ΔF represented the corresponding fluorescence intensity change). The detection limit was calculated to be 0.18 nM according to the $3\sigma/k$ principle (**Figure S8B**), which was much higher than the **H_2**-substituted nCSC system.

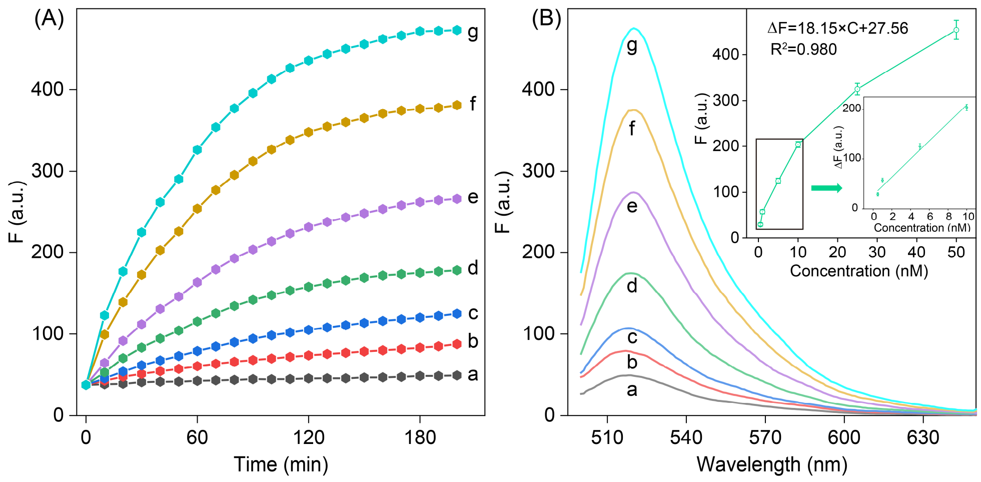

Figure S8. Performance of the CDC for miR-155 analysis. (A) Time-dependent fluorescence changes of the CDC amplifier incubated with different concentrations of miR-155 : (a) 0 nM, (b) 0.5 nM, (c) 1 nM, (d) 5 nM, (e) 10 nM, (f) 25 nM, and (g) 50 nM. (B) The corresponding fluorescence spectra as shown in S8A at 180 min. Inset: the calibration curve as indicated by the fluorescence intensity change (λ=520) as a function of the miR-155 concentration. Error bars indicate the mean ± SD of three biological replicates.

Stability of the CSC circuitry in diluted serum samples

The performance of the CSC system in diluted human serum was evaluated (**Figure S9**). The fluorescence readout even in 15% serum (ser) solution was comparable to that in ideal buffer solution, indicating high stability and acceptable accuracy of the CSC circuitry in complicated biological samples.

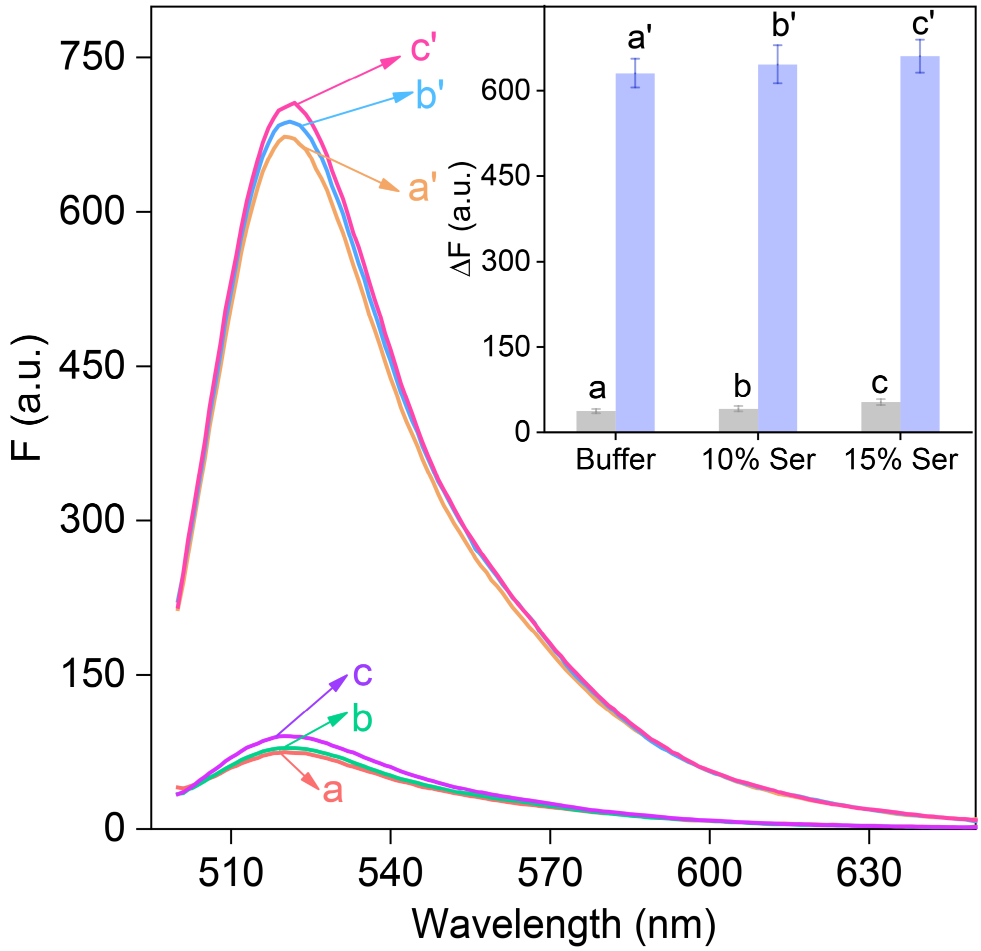

Figure S9. Fluorescence spectra of the CSC system in different reaction solutions: buffer only (a), buffer with 10 nM miR-155 (a’), 10% serum only (b), 10% serum with 10 nM miR-155 (b’), 15% serum only (c), 15% serum with 10 nM miR-155 (c’). Inset: corresponding statistical histograms of the fluorescence intensity changes (at λ = 520 nm). Error bars indicate the mean ± SD of three biological replicates.

The sensing performance of the phosphorothioated CSC system

To improve stability in a complex cellular environment, all CSC probes were partially modified with phosphorothioate bonds. The fluorescence experiments were performed to explore the signal transduction performance of the phosphorothioated CSC probes. As shown in **Figure S10**, the phosphorothioated CSC mixtures observed no obvious fluorescence signal without the miR-155 promotor (curve a), while a significantly enhanced fluorescence response was observed upon its incubation with the miR-155 promotor (curve a'), demonstrating miRNA-stimulated assembly performance. The fluorescence signal of the phosphorothioated DNA probes coincides with the unmodified CSC probe, indicating that the introduction of phosphorothioated probes does not affect the sensing performance of our CSC system.

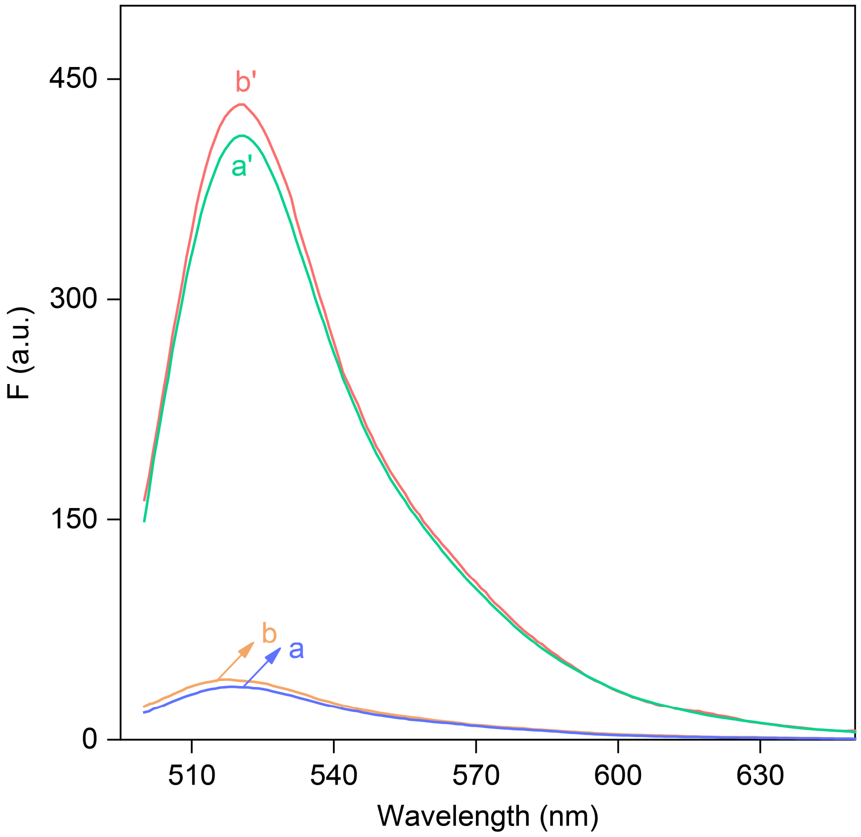

Figure S10. Fluorescence spectra generated by the phosphorothioated CSC probes with (curve a') or without (curve a) 0.5 nM miR-155 and the unmodified CSC probes with (curve b') or without (curve b) 0.5 nM miR-155.

Cytotoxicity evaluation of the CSC system

The cytotoxicity analysis toward normal cells (MCF-10A cells) and cancer cells (MCF-7) was introduced to demonstrate the favorable biocompatibility and biosafety of our CSC amplifier. As shown in **Figure S11A**, the viability of the CSC-treated cells was no less than 90% even with a high dose of DNA reactant (500 nM). Meanwhile, the viability of MCF-7 and MCF-10A cells remained high even when treated with 500 nM of the CSC system after 36 h (**Figure S11B**), indicating the favorable biocompatibility and biosafety of our designed lipo3000-loaded CSC amplifier.

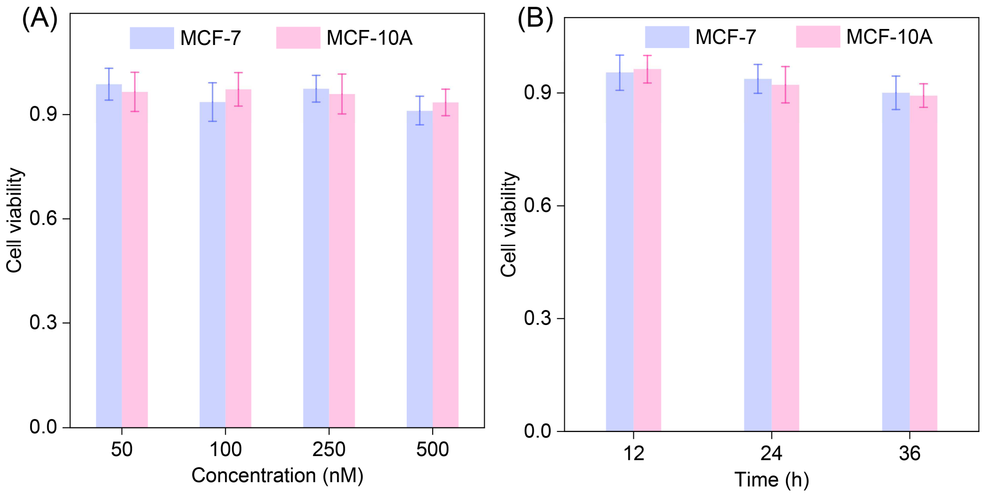

Figure S11. **Cytotoxicity evaluation of the CSC system.** (A) Cell viability of MCF-7 and MCF-10A cells incubated with various concentrations of the CSC reactants for 24 h, respectively. (B) Cell viability of MCF-7 and MCF-10A cells after various incubation times with 500 nM CSC reactants. Error bars indicate the mean ± SD of five biological replicates.

Optimization of incubation time

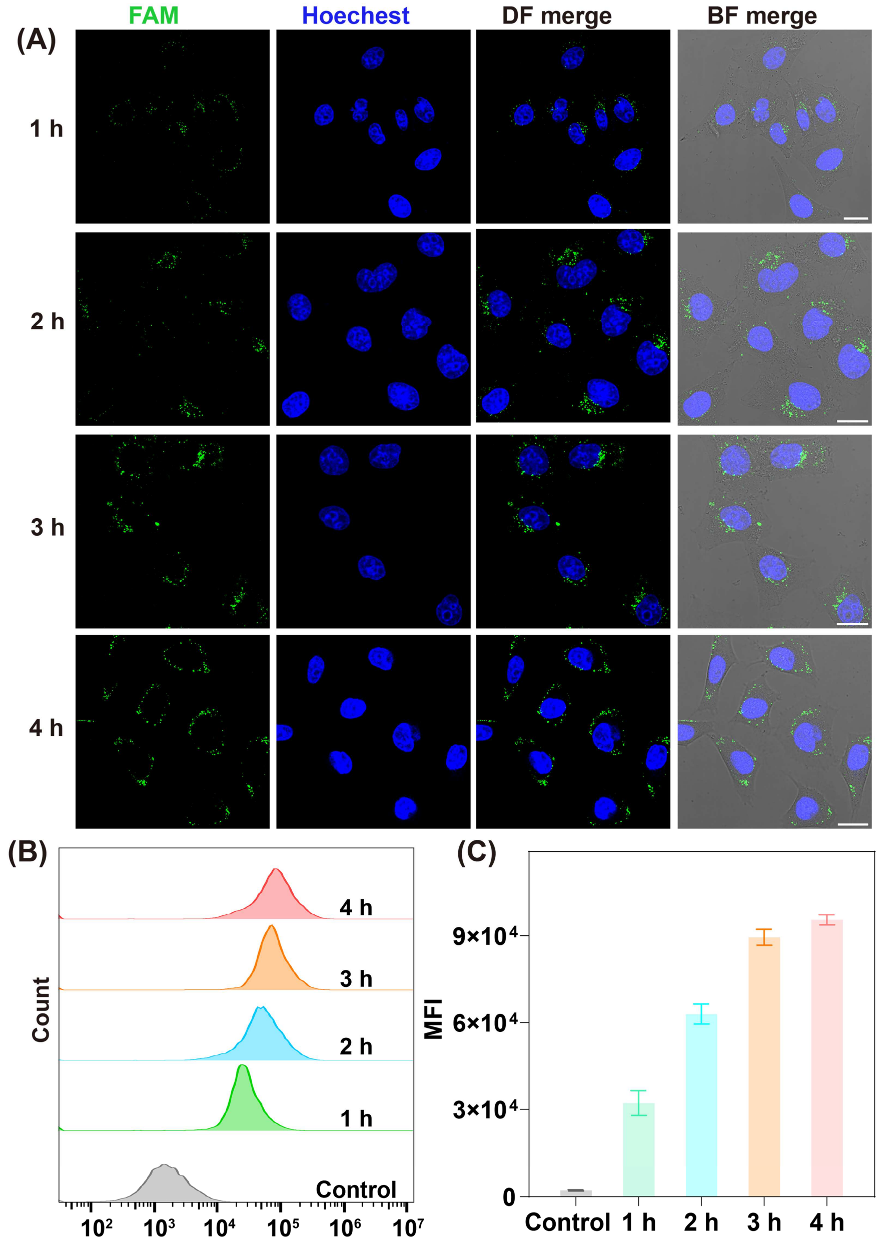

Figure S12. Time-dependent performance of the CSC amplifier in live MCF-7 cells. (A) Confocal laser scanning microscopy (CLSM) imaging and (B) Flow cytometry analysis of fluorescence intensity at different time durations in live MCF-7 cells. (C) The corresponding mean fluorescence intensity (MFI) in Figure S12B. All scale bars correspond to 20 μm. Error bars indicate the mean ± SD of three biological replicates.

The intracellular fluorescence signal of the miRNA-initiated CSC system

The sensing performance of the CSC system was also investigated by quantitative flow cytometry assay. An intense fluorescence response was observed in CSC-treated MCF-7 cells as compared to that of the nCSC-treated MCF-7 cells and the CDC-treated MCF-7 cells (**Figure S13A**), demonstrating the enhanced signal amplification features of the proposed CSC system inside living cells. However, faint fluorescence signal was observed in the miR-155 inhibitor-pretreated MCF-7 cells (**Figure S13B**), confirming that the fluorescence signal was indeed generated by the miR-155-initiated concatenated hybridization reaction and the designed CSC system is well-adapted for determinating miRNA of low concentration in living cells. The quantitative flow cytometry assay results were highly consistent with the CLSM observation, demonstrating that our proposed CSC amplifier is, indeed, suitable for *in situ* detection of less-abundantly expressed miRNAs.

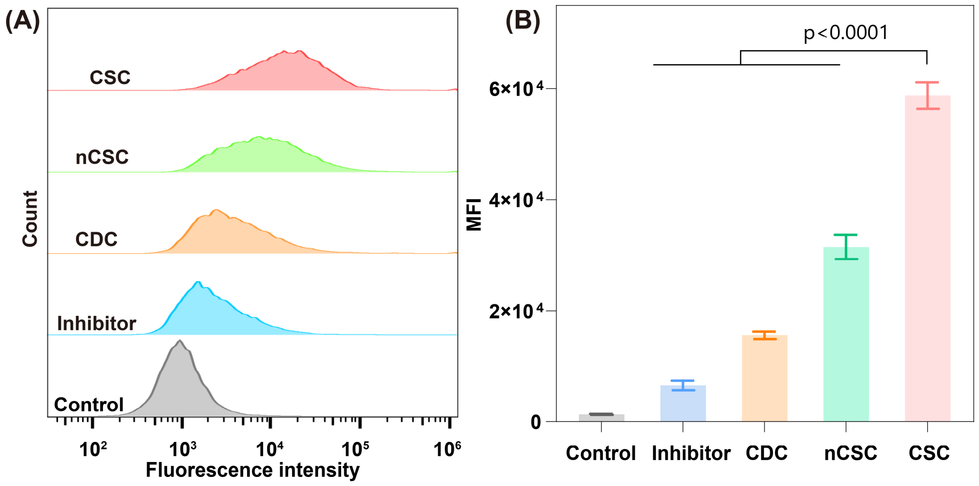

Figure S13. (A) Flow cytometric analysis and corresponding statistical histogram analysis of the mean fluorescence intensity (MFI) of the four groups in MCF-7 cells with different treatments. one-way ANOVA test. Error bars indicate the mean ± SD of three biological replicates.

The robustness of the modified DNA probe in different cell types

To explore the stability of our CSC system, the transfection efficiency of lipofectamine 3000-loaded DNA probes in MCF-7, HeLa, HEK-293T, and MCF-10A cells have been investigated. After incubation of lipofectamine 3000-loaded FAM-labeled DNA for 3 h, the most intense fluorescence signal was detected in HeLa and HEK-293T cells, yet a relatively weaker fluorescence signal was observed in MCF-7 and MCF-10A cells (**Figure S14A**). According to the statistical histogram analysis in **Figure S14B**, HeLa and HEK-293T cells observed high transfection efficiency while MCF-7 and MCF-10A cells exhibited moderate transfection efficiency, which was consistent with manufactures references [https://www.thermofisher.cn/cn/zh/home/brands/product-brand/lipofectamine/lipofectamine-3000.html#enhance]. In addition, scarcely any difference in fluorescence readout was recorded for the lipofectamine 3000-loaded FAM/BHQ-1 modified DNA-treated MCF-7, HeLa, HEK-293T, and MCF-10A cells, demonstrating the robustness of the DNA probe in complex intracellular environment.

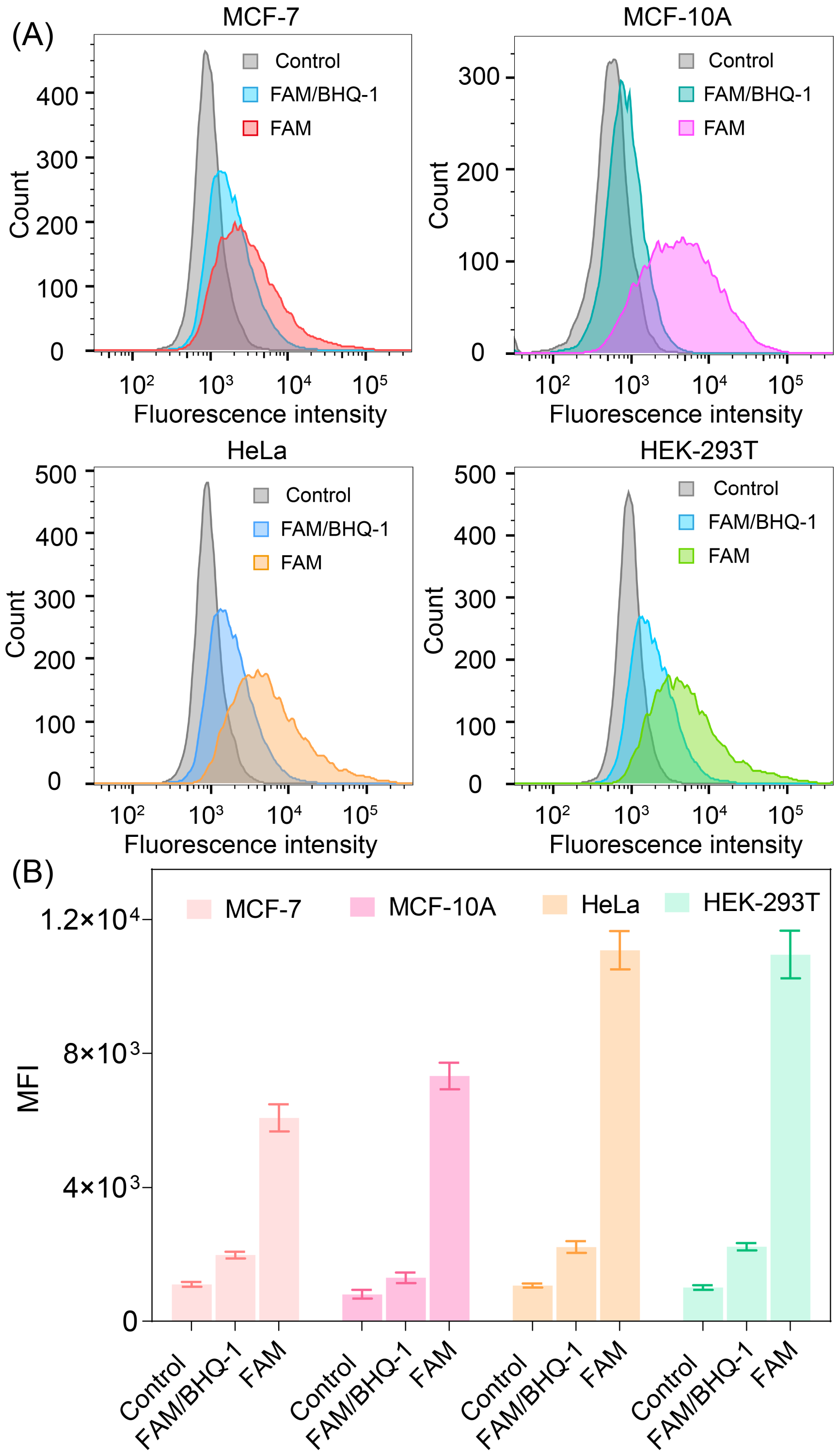

Figure S14. Flow cytometry assay the robustness of the DNA probe in different cells. (A) Flow cytometric analysis and (B) corresponding statistical histogram analysis of the mean fluorescence intensity (MFI) of the MCF-7, MCF-10A, HeLa, and HEK-293T cells with different treatments for 3 h. Error bars indicate the mean ± SD of three biological replicates.

The miRNA-specific distinction of different cells

The relative fluorescence efficiency of the flow cytometric analysis (**Figure 5C**) is calculated in comparison to the MCF-7 cells. As shown in **Figure S15**, the MCF-7 cells exhibited 2.4-fold higher fluorescence response than the HeLa cells, 7.7-fold higher fluorescence readout than the HEK-293T cells, and 9.4-fold higher fluorescence intensity than the MCF-10A cells. Compared to the MCF-7 cells with relatively lower transfection efficiency, HeLa cells with higher transfection efficiency observed weaker fluorescence response, demonstrating the relatively low miR-155 expression in HeLa cells. By contrast, almost no detectable fluorescence readout was observed in normal MCF-10A and HEK-293T cells, indicating a comparatively higher miR-155 content in tumour cells, not in normal cells. These results demonstrated that the designed CSC system was able to differentiate different cell types with diverse miR-155 content.

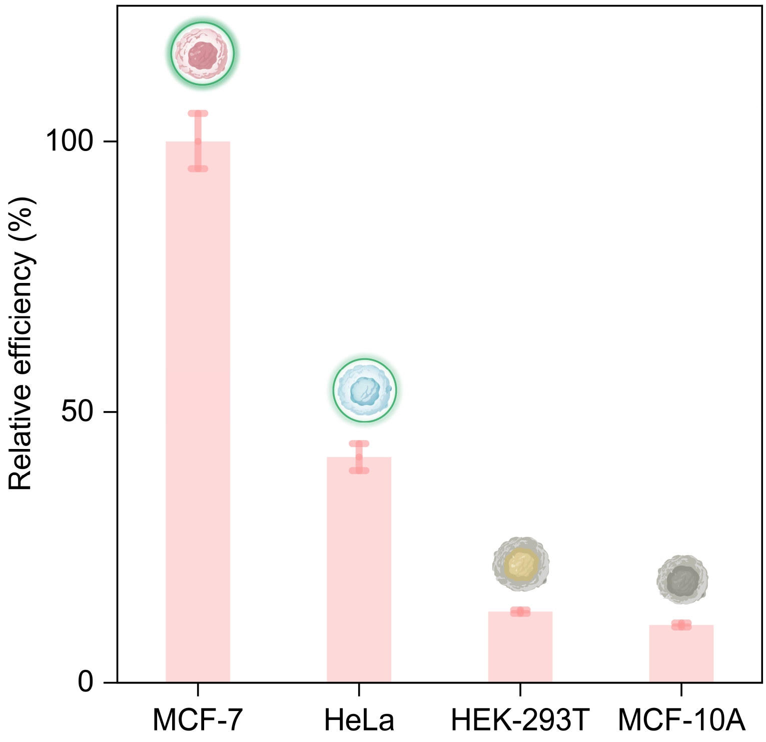

Figure S15. The relative fluorescence intensity efficiency of different cells as compared to the MCF-7 cells. Error bars indicate the mean ± SD of three biological replicates.

The qRT-PCR analysis of miR-155 expression in different cells

The gold standard quantitative reverse transcription-PCR (qRT-PCR) analysis was performed to evaluate the miR-155 expression levels in MCF-7, HeLa, HEK-293T, and MCF-10A cells. According to the results shown in **Figure S16**, the relative miR-155 expression level of MCF-7 is higher than that of HeLa, and the miR-155 content of HeLa is higher than that of HEK-293T and MCF-10A. The qRT-PCR results were consistent with that of CLSM observation and flow cytometry analysis, implying the capability of our designed CSC system for reliable discrimination of living cells with diverse miR-155 content. Therefore, it was endogenous miR-155 that initiated the concatenated hybridization process to generate the fluorescence signal and the proposed CSC imaging amplifier was able to differentiate different cell types based on the varied expression levels of intracellular miR-155.

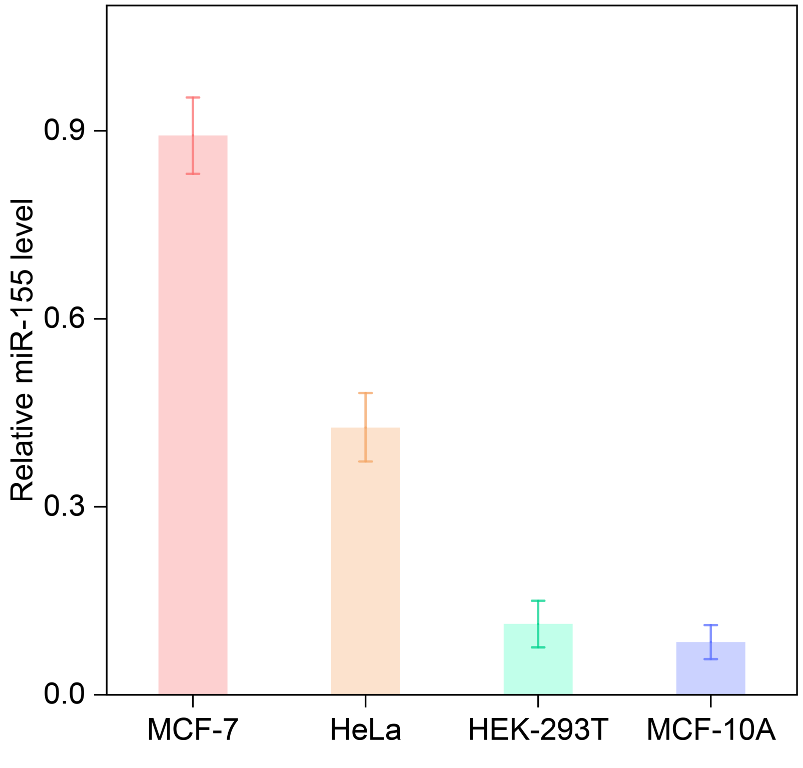

Figure S16. The relative expressions of miR-155 in MCF-7, HeLa, HEK-293T, and MCF-10A cells by using gold standard qRT-PCR analysis. Error bars indicate the mean ± SD of five biological replicates.

Hemolytic analysis of the CSC system

A hemocompatibility assay was performed by incubating various concentrations of the lipofectamine 3000-loaded CSC system in 2% red blood cells at 37 °C for 4 h. As shown in **Figure S17**, scarcely any hemolytic reaction was observed, indicating the good compatibility of the CSC system.

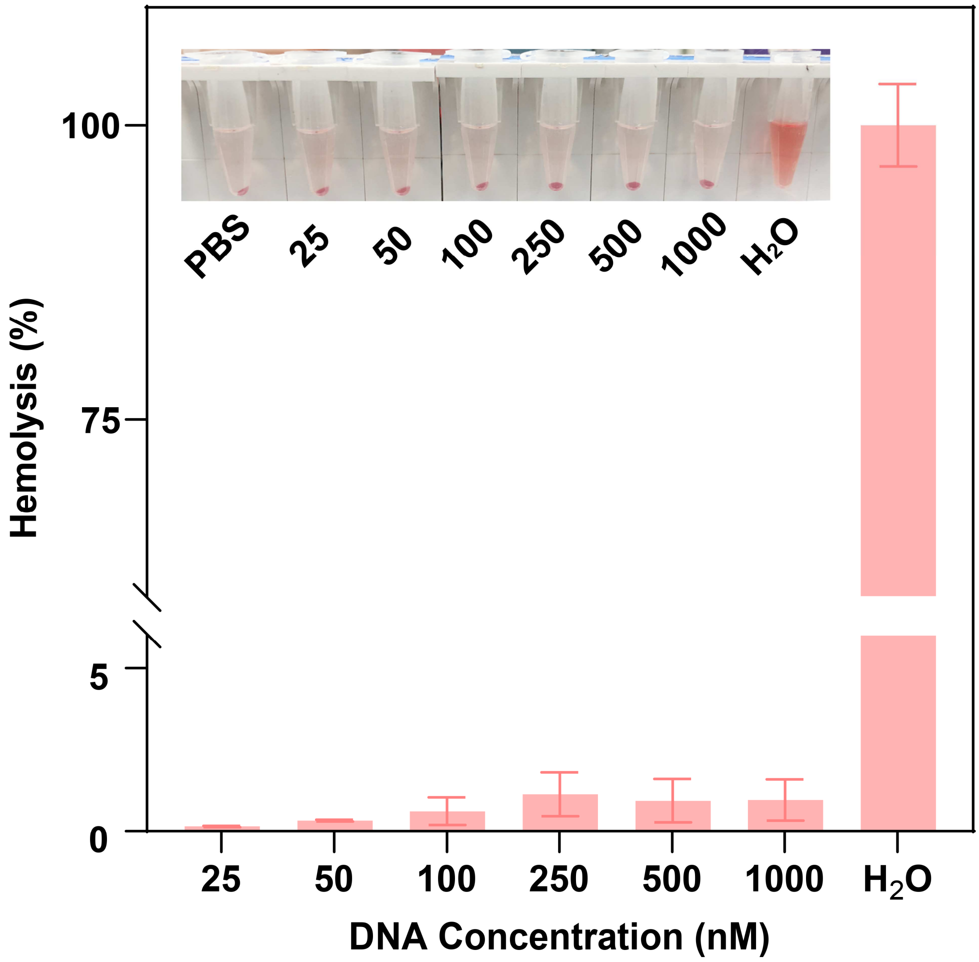

Figure S17. Hemolytic analysis of lipofectamine 3000-loaded CSC amplifier at varied concentrations. PBS and H_2O were used as the positive and negative control, respectively. Error bars presented mean ± SD of five independent replicates.

Ex vivo fluorescence imaging

The ex vivo imaging of harvested tumours and major organs was evaluated after 24 h post-injection. As shown in **Figure S18**, the Cy5 fluorescence of normal organs observed no obvious difference for all groups, indicating the high availability of CSC system for effective and robust miRNA imaging in vivo.

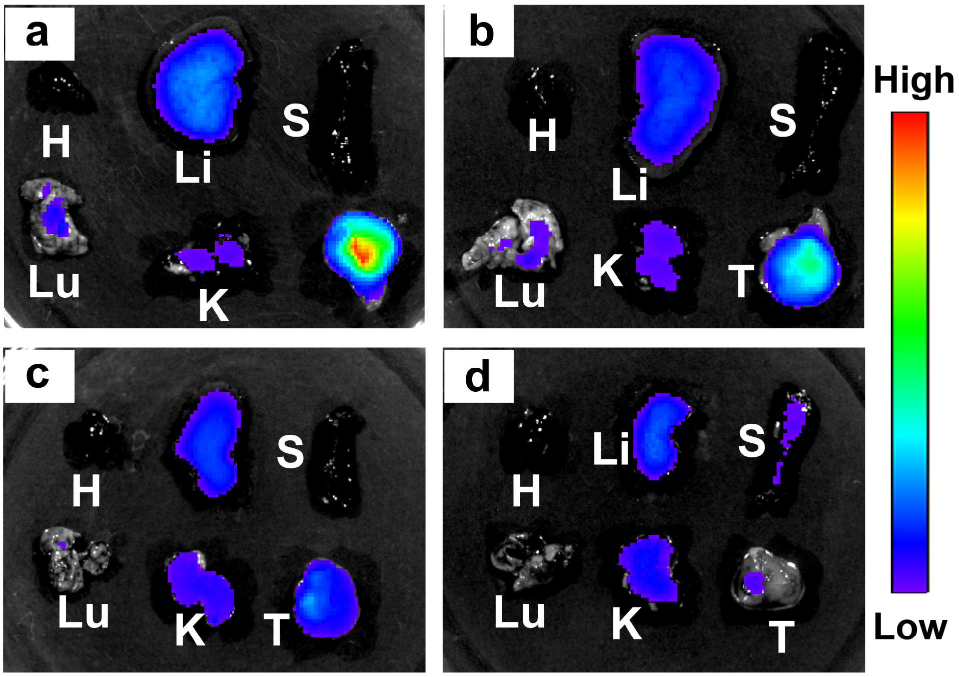

Figure S18. *Ex vivo* fluorescent images of major tissues and tumours after (a) intact CSC system, (b) nCSC system, (c) CDC system, and (d) anti-miR-155-pretreated CSC system administration for 24 h. H: heart, Li: liver, S: spleen, Lu: lung, and K: kidney, T: tumour.

Hematology and biochemical analyses of the CSC system

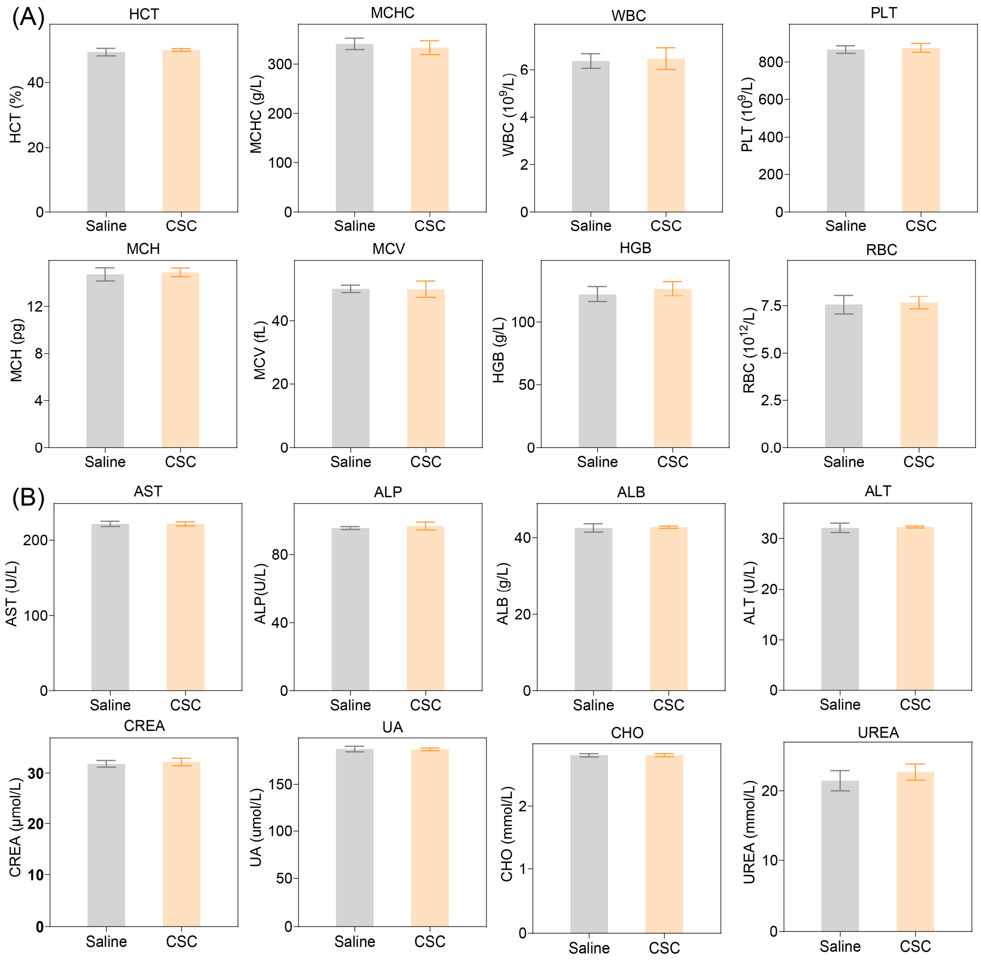

Figure S19. Biosafety evaluation of the lipofectamine 3000-loaded CSC *in vivo*. (A) Whole blood cell analysis of the mice after 24 h post intravenous injection lipofectamine 3000-loaded CSC amplifier. The tested indexes include hematocrit (HCT), mean corpuscular hemoglobin concentration (MCHC), white blood cells (WBC), platelets (PLT), mean corpuscular hemoglobin (MCH), mean corpuscular volume (MCV), hemoglobin (HGB), and blood cells (RBC). (B) Hepatic and renal functions analysis of the intravenously injected mice with lipofectamine 3000-loaded CSC after 24 h. The tested indexes include aspartate aminotransferase (AST), alkaline phosphatase (ALP), albumin (ALB), alanine aminotransferase (ALT), creatinine (CREA), uric acid (UA), cholesterol (CHO), and urea nitrogen (UREA). Error bars indicate the mean ± SD of three biological replicates.

Representative H&E-stained images of the main organs

Hematoxylin and eosin (H&E) staining was carried out to demonstrate the biocompatible of the CSC system. As shown in **Figure S20**, no obvious inflammation or disorganization was observed in these main organs in lipofectamine 3000-loaded CSC-treated mice, indicating the satisfactory histocompatibility of our CSC system.

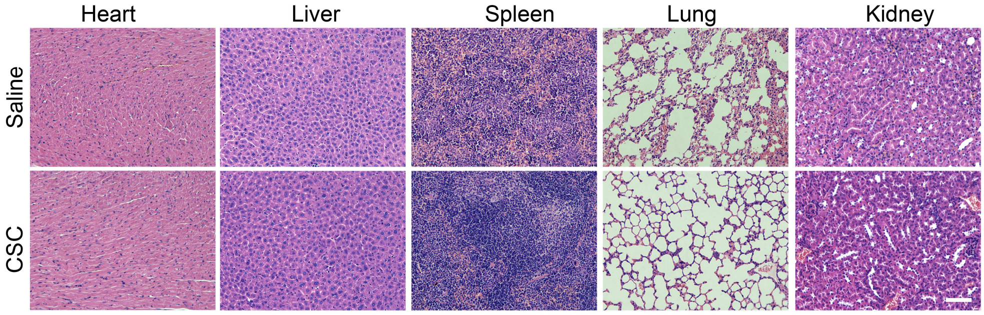

Figure S20. Biosafety investigation of the CSC system. Representative H&E-stained images of the main organs after intravenous injection with the CSC system for 24 h. The saline (PBS) administration is chosen as the control group. Scale bars are 200 μm.

Table S3. Comparison of different nucleic acid sensing methods

System	Sensing Duration(h)	Number of reactants	Sensitivity (M)	Ref.
Hybridization chain reaction based on Y-shaped DNA structure	3	6	8.0×10^{-13}	[1]
The nanoflare basis on gold nanoparticle (AuNP)	3	2	9.0×10^{-12}	[2]
Nucleic acid enzymes (MNAzymes) integrated tetrahedral DNA frameworks	1.5	8	5.4×10^{-14}	[3]
Branched catalytic hairpin assembly based on Y-scaffold DNA	6	6	2.5×10^{-9}	[4]
Proximity-driven catalytic hairpin assembly on AuNPs	3	8	2.4×10^{-12}	[5]
Domino-like localized cascade toehold assembly	3	4	5.1×10^{-11}	[6]
Hierarchical self-assembly of DNA nanostructure by catalytic self-defined circuit	3	3	2.5×10^{-13}	This work

References

[1] H. Wu, T. Chem, X. Wang, Y. Ke, J. Jiang, *Chem. Sci.*, **2020**, 11, 62-69.

[2] J. Li, J. Wang, S. Liu, N. Xie, K. Quan, Y. Yang, X. Yang, J. Huang, K. Wang, *Angew. Chem., Int. Ed.*, **2020**, 59, 20104-20111.

[3] D. Zhu, Y. Wei, T. Sun, C. Zhang, L. Ang, S. Su, X. Mao, Q. Li, C. Fan, X. Zuo, J. Chao, L. Wang, *Anal. Chem.*, **2021**, 93, 2226-2234.

[4] S. Yue, X. Song, W. Song, S. Bi, *Chem. Sci.*, **2019**, 10, 1651-1658.

[5] Z. Huang, X. Ma, F. Jiang, R. Wang, Z. Wu, Y. Lu, *Nano Lett.*, **2023**, 23, 6042-6049.

[6] Z. Yang, B. Liu, T. Huang, M. Sun, W. J. Duan, M. M. Li, J.X. Chen, Z. Dai, J. Chen, *Chem. Sci.*, **2022**, 13, 14373-14381.
